# Differential cytokine architecture in patients treated with CART19 versus CART22

**DOI:** 10.1136/jitc-2026-015126

**Published:** 2026-06-19

**Authors:** Caroline Diorio, Rawan Shraim, Anusha Thadi, Anastasia Frank-Kamenetskii, Lahari Uppuluri, Hannah Klinghoffer, Zachary Martinez, Apoorva Babu, Regina M Myers, Joseph A Fraietta, Andrew Hughes, Jonathan Sussman, Jason Xu, Susan E McClory, Katherine Mueller, Jessica Perazzelli, Laura A Vella, Sarah E Henrickson, Marco Ruella, Edward M Behrens, Amanda M DiNofia, Janis K Burkhardt, Shannon L Maude, Scott Canna, Kai Tan, Stephan Grupp, David T Teachey

**Affiliations:** 1 Division of Oncology, Children’s Hospital of Philadelphia, Philadelphia, Pennsylvania, USA; 2 Abramson Cancer Center, University of Pennsylvania, Philadelphia, Pennsylvania, USA; 3 Immune Dysregulation Program, Children’s Hospital of Philadelphia, Philadelphia, Pennsylvania, USA; 4 Division of Oncology and Center for Childhood Cancer Research, The Children’s Hospital of Philadelphia, Philadelphia, Pennsylvania, USA; 5 Department of Biomedical and Health Informatics, The Children’s Hospital of Philadelphia, Philadelphia, Pennsylvania, USA; 6 Center for Cellular Immunotherapies, University of Pennsylvania, Philadelphia, Pennsylvania, USA; 7 Division of Infectious Diseases, Children's Hospital of Philadelphia, Philadelphia, Pennsylvania, USA; 8 Division of Immunology, Children’s Hospital of Philadelphia, Philadelphia, Pennsylvania, USA; 9 Division of Rheumatology, Children’s Hospital of Philadelphia, Philadelphia, Pennsylvania, USA; 10 Pathology and Laboratory Medicine, University of Pennsylvania Perelman School of Medicine, Philadelphia, Pennsylvania, USA

**Keywords:** Cytokine, Cytokine release syndrome, Chimeric antigen receptor - CAR

## Abstract

**Background:**

Cytokine release syndrome (CRS) is a life-threatening toxicity of chimeric antigen receptor T-cell therapy (CART) for B-cell acute lymphoblastic leukemia (B-ALL). Lower rates of severe CRS have been reported in patients treated with CD22-directed CART (CART22) compared with those treated with CD19-directed CART (CART19).

**Methods:**

More than 1,000 serum proteins were measured using a proximity extension assay on 40 patients treated with CART19 or CART22 and cytokines were compared. Single-cell (single-cell RNA-sequencing (scRNAseq)) was used to identify cellular and transcriptional differences between patients treated with CART19 and CART22. CART19-blast and CART22-blast interactions at the immune synapse (IS) were modeled in vitro.

**Results:**

We identified interleukin-10 (IL-10) as a critical endogenous modulator of CRS and demonstrated that previous treatment with CART is associated with increased serum IL-10 in the setting of subsequent relapse. Mechanistically, IL-10 induced higher interferon gamma expression and *SOCS3* upregulation in both CART19 and CART22 cells. IL-10 differentially impacted the CART IS, prolonging the CART19 but shortening the CART22 IS. Using scRNAseq we demonstrated upregulation of *SOCS3* in CART22 patient CD4 T-cells, potentially suppressing trans-IL-6 signaling.

**Conclusions:**

These findings reveal IL-10 as a potent CRS‐mitigating factor and support IL-10 enhancement strategies to improve the safety of CART.

**WHAT IS ALREADY KNOWN ON THIS TOPIC**

- Cytokine release syndrome (CRS) has been well characterized following CD19-directed chimeric antigen receptor T-cell (CART19) in B-cell acute lymphoblastic leukemia (B-ALL). Less is known about CRS following CD22-directed CART (CART22) for B-ALL.

**WHAT THIS STUDY ADDS**

- We comprehensively examine cytokine architecture following CART22 treatment compared with CART19 treatment. Despite high disease burden, patients treated with CART22 did not experience severe CRS. We identify preinfusion and postinfusion differences in serum interleukin-10 (IL-10) levels as a potential contributor to this phenomenon.

**HOW THIS STUDY MIGHT AFFECT RESEARCH, PRACTICE OR POLICY**

- We identify IL-10 as a novel, potentially modifiable association with lower-grade CRS.

## Introduction

 Cytokine release syndrome (CRS) is the most common serious adverse event following therapy with chimeric antigen receptor T-cells (CARTs). CRS occurs on a spectrum of severity from isolated fevers to hypotension and end organ dysfunction.^1^ CRS results from excessive and disproportionate activation of immune cells leading to high levels of interleukin (IL)-6, IL-1, and interferon gamma (IFNγ).^2^ IL-6 is an accepted driver of CRS pathophysiology, and blockade of IL-6R with tocilizumab is the standard therapy for the treatment of moderate and severe CRS.^3^ Many cytokines are elevated in the setting of CRS, and the role of the majority of these cytokines has not been fully elucidated. The bulk of experience with treatment of severe CRS, and our understanding of the biology of CRS comes from CD19-directed CART (CART19) in hematologic malignancies. In patients with leukemia, prior reports by multiple groups have found higher disease burden, often defined as a marrow burden of at least 25% or higher, is the most consistently identified risk factor for severe CRS, with rates of severe (Grade 3 or higher) CRS exceeding 30% in patients with high disease burden.^4^,^7^ Rates of severe CRS have decreased over time as patients are increasingly treated with CART at lower disease burden and as anti-IL-6 therapies are used earlier in treatment.^8^,^10^

Although CART19 is highly effective in inducing remission, approximately 50% of patients subsequently relapse, with 1/3 of relapses associated with CD19-negative antigen escape.^11^ Targeting CD22 is a promising strategy for patients with B-cell acute lymphoblastic leukemia (B-ALL) who relapse with CD19-negative disease. We recently demonstrated the efficacy of a CD22-directed CART (CART22) in a cohort of heavily pretreated patients with high leukemic disease burden and CD19-negative disease.^12^ Despite high disease burden, no patients on this trial developed severe CRS.^12^ This low rate of severe CRS was similar to reports from other CART22 studies.^13 14^ Across all trials of CART22 to date, most patients had previously received a CD19-directed immunotherapy (CART19 or blinatumomab).

We hypothesized that the distinct toxicity profile observed in CART22 patients compared with CART19 patients may be due to host microenvironmental factors. We sought to identify the host and CART-mediated factors contributing to this surprising lack of severe toxicity despite high disease burden in CART22 patients. We compared a cohort of patients treated with CART19 and CART22 and combined deep immunophenotyping on patient samples with in vitro immune synapse (IS) quantitation assays. We identified serum proteomic, cellular, and transcriptional factors that may mitigate expected toxicity.

## Methods

### Included patients and disease definitions

Patients were treated with a 4–1BB stimulated CART19 CTL019 on NCT01626495 or NCT02906371 or a 4–1BB stimulated CART22 (CART22-65s) on NCT02650414. Patients who received CART19 were purposively sampled to include both minimal and severe CRS cases. All patients treated with CART22 on the initially published clinical trial were included.^12^ CRS was graded according to the American Society for Transplantation and Cellular Therapy (ASTCT) consensus criteria.^1^ Minimal CRS was defined as no CRS or Grade 1 or 2 CRS. Severe CRS was defined as Grade 3 or higher CRS. Disease burden was measured at the preinfusion disease assessment following lymphodepleting chemotherapy and was defined as the highest percentage of bone marrow blasts as measured by multiparameter flow cytometry for measurable residual disease, aspirate morphology or biopsy.

### Quantitative PCR

Quantitative PCR was performed with ABI TaqMan technology targeting specific sequences of the integrated transgene, as previously described.^12^

### Proximity extension assay experiments

Cryopreserved serum was thawed and plated for Olink experiments. Samples were randomly assigned across plates in all experiments to minimize bias. The Olink Explore 1536/384 panel (Olink Proteomics, Uppsala, Sweden) was used to measure more than 1,400 proteins in CART19 and CART22 patients.^15^ Cytokine measurements from marrow serum were determined on the Olink Signature Q100 machine (Olink, Waltham, Massachusetts, USA) using a Target 48 Cytokine Panel. Raw data (pg/mL) was log2 transformed for analysis. Pretreatment cytokine measurements from patients treated on NCT02374333 and NCT03792633 were obtained using the Target 96 Immuno-Oncology Panel (Olink) using the Signature Q100.

### CART production for in vitro assays

CART19 and CART22 were prepared as previously described.^16^ Briefly, normal donor T cells were obtained from the University of Pennsylvania Human Immunology Core and activated via CD3/CD28 Costimulatory bead activation. After 48 hours, cells were transduced with lentiviral vectors to express either the CD19-CAR or CD22-CAR construct. Following costimulatory bead removal, cells were expanded over the course of 7–10 days, and frozen in fetal bovine serum (FBS)+10% dimethyl sulfoxide once the T cells reached resting size.

### Immune synapse experiments

Live-cell confocal imaging was performed using a protocol adapted from previously described methods (online supplemental methods).^17^,^19^ IS duration was defined as the time between CART calcium flux and propidium iodide (PI) uptake by the target cell. IS events persisting for more than 30 min without PI uptake were considered unterminated. Each independent experiment included two to three replicate co-cultures per condition, and one to two IS events were quantified per co-culture. Data were pooled from three independent experiments, with each data point representing a single IS event. Cytokine production was assessed from 50 µL of supernatant collected from co-cultures of CART and target cells (NALM6 or UOCB) at a 1:3 effector-to-target (E:T) ratio. IFNγ levels were quantified using Cytometric Bead Array Flex Sets (BD Biosciences), following the manufacturer’s instructions. Samples were analyzed using a CytoFLEX5 flow cytometer (Beckman Coulter).

### Co-culture experiments

NALM6 or UOCB cell lines transduced to express GFP were co-cultured with CART cells at an E:T ratio of 1:8 in RPMI supplemented with 20% FBS and 1% penicillin/streptomycin, 1% HEPES, 1% non-essential amino acids, and 1% L-glutamine. Cells were incubated with or without IL-10 (Thermo Fisher, Cat# 200–10-100UG) added at a concentration of 15 ng/mL. After incubating for 24 hours cytotoxicity was assessed by luciferase assay (Promega) following the manufacturer’s instructions. In a separate experiment, cells were washed in phosphate-buffered saline and then snap frozen on dry ice. All experiments were performed in triplicate.

### Bulk RNA sequencing

RNA was extracted from cryopreserved pellets using Qiagen’s QiaShredder (Cat no. 79656) and RNeasy Mini RNA extraction kit (Cat no. 74104). Complementary DNA libraries were prepared with Illumina’s Stranded messenger RNA poly-A enrichment kit. Pooled libraries were sequenced on NextSeq2000 on a P4 XLEAP SBS flow cell to collect 20 million reads per sample.

### Single-cell RNA sequencing

Cryopreserved peripheral blood mononuclear cells (PBMCs) or bone marrow mononuclear cells (BMMCs) were thawed. Hashed and pooled cell suspensions or individual cell suspensions were subsequently processed to generate single-cell RNA-sequencing (scRNAseq) libraries using chromium Next GEM single cell 3’ reagent kits V.3.1 Dual Index (10x Genomics, PN-1000268) with 3’ feature barcode technology (10x Genomics, PN-1000262) for cell surface as per manufacturer’s instructions. After pooling, libraries were sequenced on an Illumina NovaSeq 6000.

### Data analysis

All data analyses were completed in R V.4.3.3 or GraphPad Prism (versions 8–10). Code is available at github.com. See online supplemental methods for details.

### Data availability

Bulk RNA-sequencing and scRNAseq data generated by this study were deposited in Gene Expression Omnibus (GEO; GSE309375). Processed bulk RNA-sequencing data from Therapeutically Applicable Research to Generate Effective Treatments (TARGET) dataset were downloaded from the GDC portal (https://portal.gdc.cancer.gov/).

## Results

### CART22 patients experienced only minimal CRS despite high disease burden and excellent expansion of CART cells

**Figure 1:**
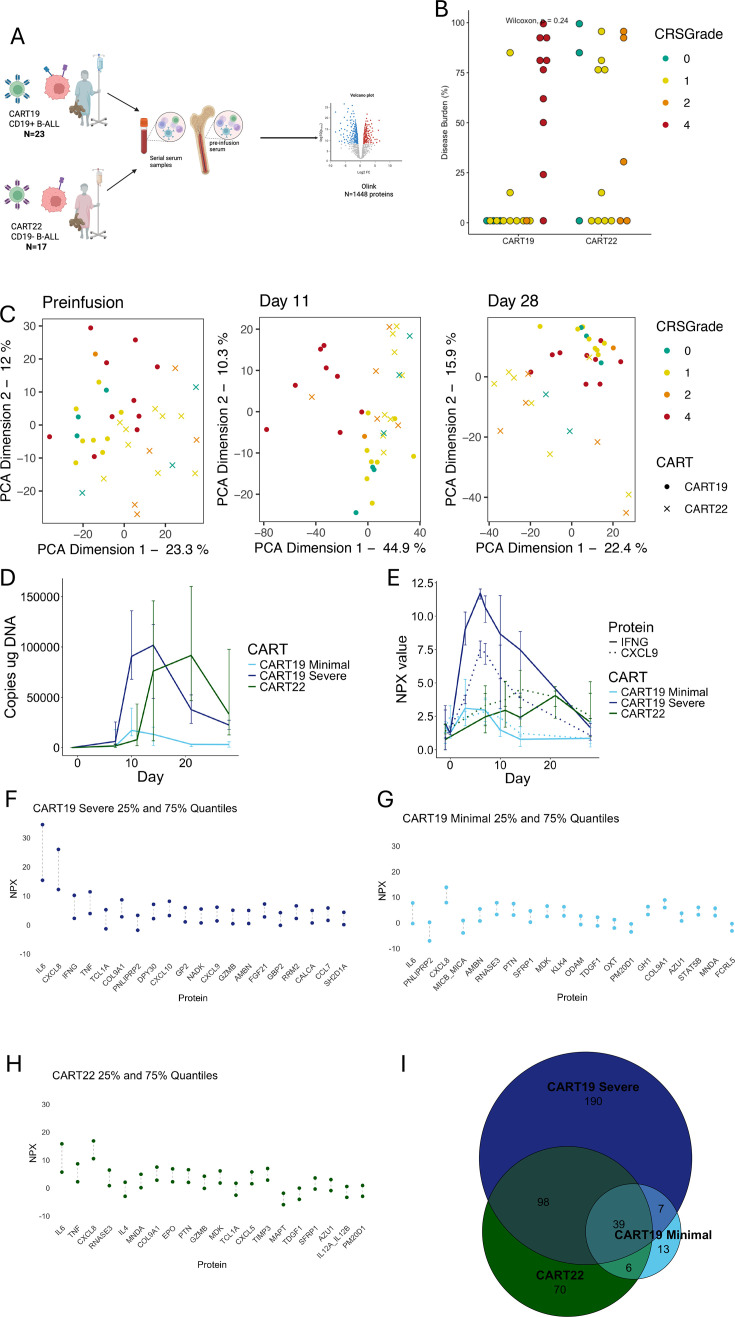
Patients treated with CART22 did not develop severe CRS despite high disease burden. (**A**) Schematic representation of experimental process. Patients treated with either CART19 (n=23) or CART22 (n=17) were included. Olink PEA was performed on serial serum samples. (**B**) Pretreatment disease burden (y-axis) in patients treated with CART19 (n=23) or CART22 (n=17). Dots are colored by grade of CRS: green—no CRS, yellow—Grade 1 CRS, orange—Grade 2 CRS and red—Grade 4 CRS. P value was calculated using the Wilcoxon test. (**C**) PCA of CART19 (circle) and CART22 (x) patients at the preinfusion, Day 11, and Day 28 time points. The percentage of the total variance explained by each principal component is shown. (**D**) Median CART quantified by qPCR in patients treated with CART22 (n=17), and CART19 with minimal CRS (n=13) or severe CRS (n=10). Error bars represent IQR. (**E**) NPX values of median IFNγ (solid line) and CXCL9 (dotted line) in CART19 Severe patients (n=23; Severe n=10, Minimal n=13) and CART22 patients (n=17). A linear mixed-effects model was applied showing a significant difference (p<0.0001) in CART group×time (Day) interaction across both IFNγ and CXCL9 NPX measurements. Error bars represent IQR. Top 20 proteins with the highest IQR in patients with (**F**) severe CRS following CART19 (n=13) (**G**) minimal CRS following CART19 (n=10) and (**H**) following CART22 (n=17). Euler diagram demonstrating the number of proteins with an IQR >2 in patients with severe or minimal CRS post CART19 or CART22 therapy (**I**). B-ALL, B-cell acute lymphoblastic leukemia; CART, chimeric antigen receptor T-cell; CART19, CD19-directed CART; CART22, CD22-directed CART; CRS, cytokine release syndrome; FC, fold change; IFNγ, interferon gamma; IL, interleukin; NPX, normalized protein expression; PCA, principal component analysis; PEA, proximity extension assay; qPCR, quantitative PCR.

We performed the proximity extension assay Olink to measure serum proteins on patients who received CART19 (n=23, CTL019 on the early phase trials NCT01626495 or NCT02906371) or CART22 (n=17, CART22-65s on NCT02650414) for relapsed/refractory B-ALL (figure 1A; online supplemental table 1). All patients treated with CART22 from the reported trial were included (n=17) and no patients developed severe CRS. The CART19 cohort was purposively sampled to include both minimal (n=13) and severe (n=10) CRS cases; the rate of severe CRS on the primary CART19 trials was 27% (8/25) and 9% (6/70) respectively.^5 6^ The CART19 and CART22 cohorts were similar, except all patients treated with CART22 had CD19-negative B-ALL and had previously received a CD19-directed immunotherapy (CART19 n=16, blinatumomab n=4; online supplemental table 1). No patients in either cohort developed immune effector cell-associated hemophagocytic-like syndrome prior to Day 28.^5 6 12^ Median disease burden at the time of infusion was not different between the CART19 (median 1.3%, IQR 0–78.2%) and CART22 (median 30.5%, IQR 1.2–85%, Wilcoxon p value=0.24) cohorts; despite this CART22 patients had no severe CRS events (graded per ASTCT Grading; figure 1B).^1^

We examined 1,448 proteins, reported as normalized protein expression (NPX), a log2 transformed score. We performed principal component analysis of the data from each time point and annotated the results based on CRS grade and CART cohort (figure 1C and online supplemental figure 1). At the preinfusion time point, separation between CART22 and CART19 patients was visible. At Day 11, Grade 4 CRS, which occurred only in CART19 patients, clustered separately. Among patients with low-grade CRS, CART19 and CART22 patients clustered separately. This pattern was also apparent at Day 28.

We systematically evaluated CART expansion in both cohorts. As previously reported, there was later expansion in CART22 (Day 21, median 91,674 copies CAR construct/µg DNA, IQR (46,751–160,110)) than in CART19 Minimal (Day 10, median 17,053 copies CAR construct/µg DNA, IQR (12,744–46,190)) or CART19 Severe (Day 14, median 101,768, IQR (48,989–122,327); figure 1D).^12^ Congruent with this observation, median IFNγ levels peaked later in CART22 patients (Day 21, median NPX 4.06 IQR (2.34–4.39)) than in CART19 minimal (Day 3, median NPX 3.1, IQR (2.26–5.27)) or CART19 severe patients (Day 6, median NPX 11.73, IQR (11.28–12.04); figure 1E). Levels of the IFNγ-responsive protein CXCL9 peaked contemporaneously with IFNγ levels in minimal CART19 (Day 7, median=2.86 NPX, IQR (1.87–3.87)) and severe CART19 patients (Day 6, median NPX 7.5, IQR (6.89–8.14)) and quickly decreased to baseline. However, in CART22 patients CXCL9 levels peaked (Day 14, median NPX 4.53, IQR (3.15–5.94)) prior to IFNγ levels and remained elevated for a prolonged period, implying that CXCL9 levels were being driven by a different mechanism following CART22 than CART19 (figure 1E). A linear mixed-effects model revealed a significant group×time interaction (p<0.0001), indicating that the pattern of change in protein levels over time differed between CART19 and CART22. This effect was similarly observed in the IFNγ-responsive protein CXCL10 (online supplemental figure 2).

We hypothesized that proteins that had the highest degree of change over time would be the ones most likely to be important in the pathophysiology of CRS. For example, a protein that did not change from pre-infusion to peak symptoms of CRS to resolution of CRS would be considered low variability versus a protein that increased or decreased significantly over time would be considered highly variable. We used the IQR to define this surrogate of variability within the three cohorts (CART19 minimal CRS, CART19 severe CRS and CART22) with an IQR ≥2 considered highly variable. The top 20 ranked proteins are shown for each cohort in figure 1F–H. Proteins that were highly variable across cohorts included IL-6, tumor necrosis factor-alpha (TNFɑ), CXCL8 and IFNγ (online supplemental data 1). We then sought to identify highly variable proteins unique to each cohort, to identify proteins that may be specifically associated with severe CRS. As expected, patients with CART19 severe CRS had the highest number of unique highly variable proteins (n=190 vs n=13 CART19 minimal CRS and n=70 CART22; figure 1I). The top three most highly variable proteins unique to severe CRS following CART19 were CCL7, the zymogen GP2, and the DPY30 subunit of histone methyltransferase DPY30 (online supplemental figure 3).

### IL-10 is more highly expressed in patients who previously received immunotherapy

**Figure 2:**
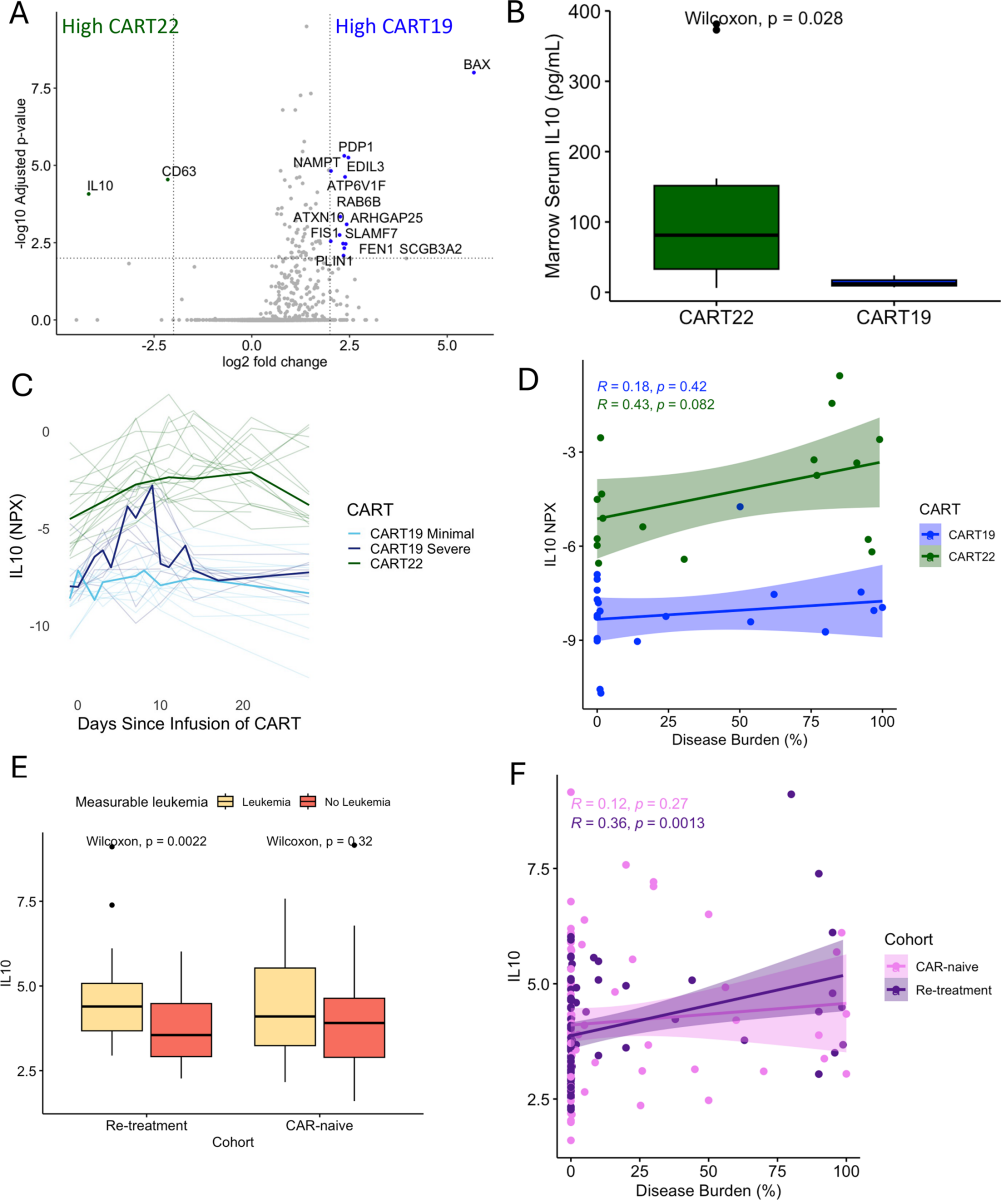
IL-10 levels are higher in CART22 patients than in CART19 patients from preinfusion to resolution. (**A**) Differentially expressed proteins between patients treated with CART22 and CART19 (minimal CRS) at the pretreatment time point measured by Olink. IL-10 was the most highly differentially expressed protein with higher levels in patients with CART22. Colored proteins are those with log2 fold change ≥2 and adjusted p value ≤0.01 (Benjamini-Hochberg). Green is up in CART22, blue is up in CART19. (**B**) Olink T-48 assay showing IL-10 levels were higher in serum from bone marrow of CART22 (n=10) patients compared with CART19 (n=9) patients. P value was calculated using the Wilcoxon test. (**C**) IL-10 levels were higher in CART22 patients (n=17) than in CART19 minimal CRS (n=13) or CART19 severe CRS (n=10) patients at every time point measured by Olink. Bold line represents median, light-colored lines represent each individual patient. (**D**) Spearman correlation between preinfusion serum IL-10 NPX level and disease burden in CART19 (n=23, Severe n=10, Minimal n=13) and CART22 (n=17) patients. (**E**) Preinfusion IL-10 levels (NPX) in patients treated on the humanized Phase I or Phase II trials on the Retreatment (n=47 MRD negative at infusion, n=29 measurable disease at infusion) or CART-naïve (n=44 MRD negative at infusion, n=37 measurable disease at infusion) cohort stratified by presence or absence of measurable leukemia at the time of infusion. P value was computed using Wilcoxon test. (**F**) Spearman correlation between preinfusion IL-10 level and disease burden in CART-naïve (n=81) or Retreatment (n=76) cohorts. CART, chimeric antigen receptor T-cell; CART19, CD19-directed CART; CART22, CD22-directed CART; CRS, cytokine release syndrome; IL, interleukin; MRD, measurable residual disease; NPX, normalized protein expression.

All patients treated with CART22 had previously been treated with CD19-directed immunotherapy. To understand if prior immunotherapy modulated the serum microenvironment, we compared differentially expressed proteins (DEPs) between CART19 and CART22 patients at the preinfusion time point. We previously demonstrated that the preinfusion serum microenvironment differs between patients with severe and minimal CRS; we therefore compared patients treated with CART22 to those treated with CART19 who went on to develop minimal CRS.^15^ IL-10 was the most highly DEP in CART22 patients and the apoptosis associated protein BAX was the most highly DEP in CART19 patients (figure 2A).^20^ The same analysis for all patients in the cohort also showed IL-10 and BAX as the most highly DEPs (online supplemental figure 4). Levels of BAX remained consistently higher over time in the CART19 cohorts compared with the CART22 cohorts (online supplemental figure 5).

A higher level of IL-10 in CART22 patients was confirmed by measuring via Luminex on preinfusion serum from CART22 patients and patients treated on the CART19 clinical trial NCT01626495. The median IL-10 levels were 26.0 pg/mL for CART22 patients (IQR 14.0–32.4, n=17) compared with 5.72 pg/mL for CART19 patients (IQR 2.13–11.5, n=30, Wilcoxon p=0.00008). Higher levels of IL-10 were also found in the marrow serum of CART22 patients compared with CART19 patients (Wilcoxon p=0.028, figure 2B). Significant differences in IL-10 family proteins were also seen, with higher levels of IL10-R1 in CART19 patients (online supplemental figure 6). IL-10 levels remained higher in CART22 than CART19 minimal or severe patients at every time point (figure 2C).

To understand if IL-10 levels were associated with the presence of B-ALL blasts, we correlated disease burden and IL-10 levels at the preinfusion time point (figure 2D). IL-10 levels tended to correlate with disease burden in CART22 patients (R=0.43, p=0.082) but not in CART19 patients (R=0.18, p=0.42). All patients in the CART22 cohort had CD19-negative B-ALL, so we next questioned whether IL-10 levels could be due to CD19-negative blasts. We compared transcriptional expression of *IL10* in blasts from the TARGET relapse cohort and found minimal *IL10* expression in general (online supplemental figure 7). We also examined a previously published scRNAseq data set that examined patients with CD19 positive disease and a subsequent CD19 negative relapse.^21^ We found essentially no transcriptional expression of *IL10* in either group (CD19 positive blasts *IL10* expression 0.001, n=16 623 blasts; CD19 negative blasts *IL10* expression 0, n=8020 blasts). We concluded that IL-10 was unlikely to be coming from the blasts.

We hypothesized that higher IL-10 levels were specific to prior CART therapy. We examined a separate cohort of patients (n=156) who were enrolled on the humanized CART19 (huCART19) Phase I (NCT02374333) or Phase II studies (NCT03792633) and received huCART19 de novo (“CAR-naïve”) or for loss of circulating CART with or without leukemia relapse (“Re-treatment”). IL-10 levels at the preinfusion time point were not different in the CAR-naïve cohort, regardless of the presence of measurable leukemia (figure 2F). However, in the retreatment patients, IL-10 levels were higher in patients with measurable leukemia than those without (p=0.002, Wilcoxon test). Similarly, IL-10 levels correlated with disease burden in Retreatment patients (R=0.36, p=0.001) but not CART-naïve patients (R=0.12, p=0.27; figure 2G). Taken together, these results imply that IL-10 levels were governed by a combination of both previous immunotherapy and relapsed leukemia, but not necessarily CD19 negative disease.

### IL-10 differentially impacts CART19 and CART22 immune synapses without impairing cytotoxicity

**Figure 3:**
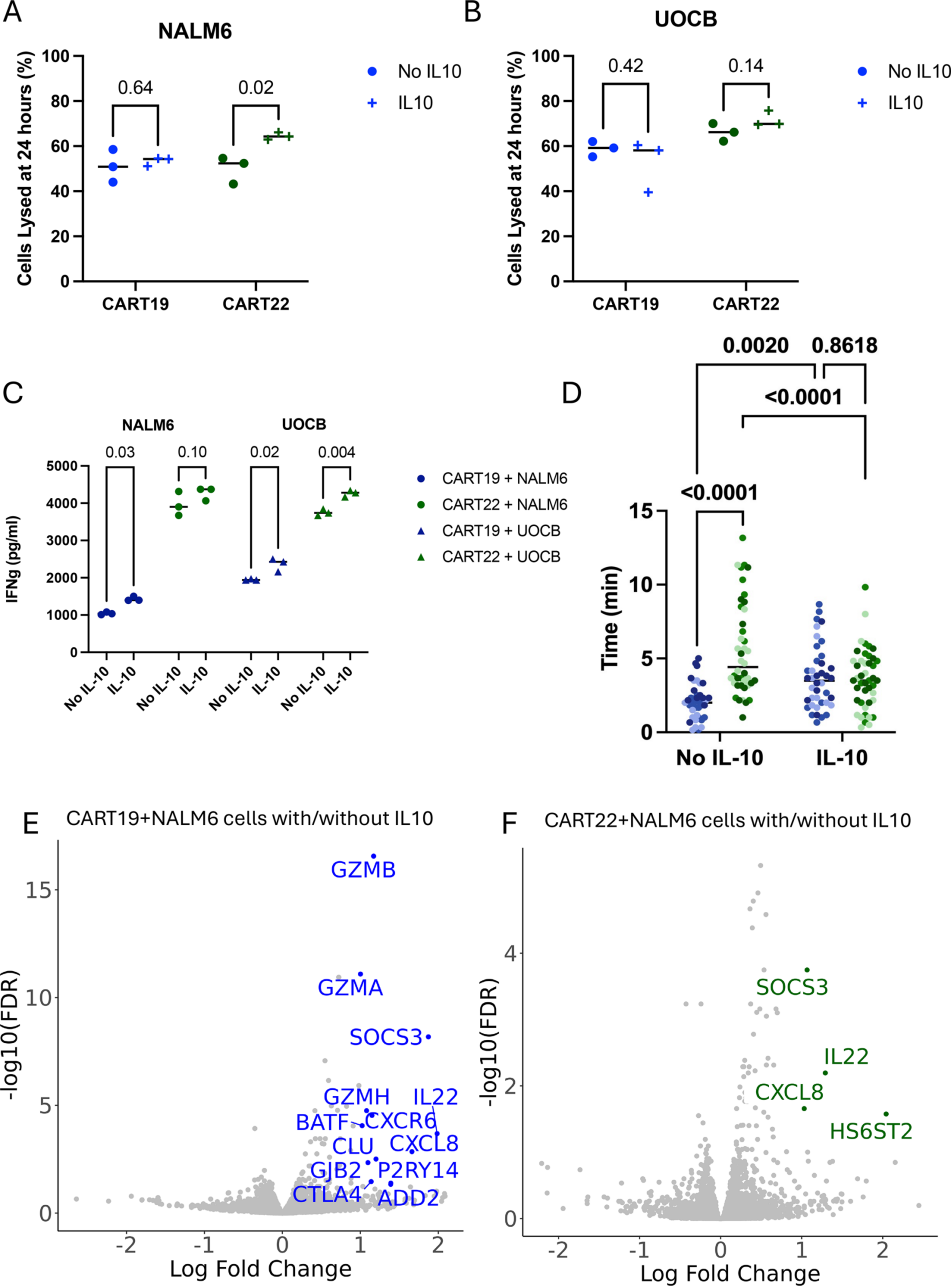
IL-10 modulates the immune synapse and interferon gamma production without impairing cytotoxicity. Cytotoxicity measured by percent lysis of cells at 24 hours in NALM6 (**A**) and UOCB (**B**) cell lines at an effector:target ratio of 1:8 with IL-10 added at 15 ng/mL. Interferon gamma (IFNγ) measured by CBA was higher when CART19 or CART22 cells were co-cultured with NALM6 or UOCB cell lines in the presence of IL-10 at 15 ng/mL. Experiments were performed at an effector:target ratio of 1:3 (**C**). All experiments were performed in triplicate. P value calculated by two-way ANOVA. Time from immune synapse initiation to termination was longer for CART22 (green) than for CART19 (blue) cells in the absence of IL-10. When IL-10 was added, the CART19-NALM6 immune synapse lengthened while the CART22-NALM6 synapse shortened (**D**). The experiment was performed with three separate donors each in triplicate. Shades represent different donors; P values were calculated by two-way ANOVA. IL-10 was added to CART19 (**E**) or CART22 (**F**) cells co-cultured with NALM6 cells at an effector:target ratio of 1:8. n=3 replicates in each condition. Differentially expressed genes (log fold change>1, FDR <0.05) are shown. ANOVA, analysis of variance; CART, chimeric antigen receptor T-cell; CART19, CD19-directed CART; CART22, CD22-directed CART; CBA, cytokine bead assay; FDR, false discovery rate; IFNγ, interferon gamma; IL, interleukin.

IL-10’s role as the canonical anti-inflammatory cytokine derives mainly from studying its effects on lipopolysaccharide (LPS)-stimulated myeloid cells.^22^ We sought to understand the role of IL-10 in CART cells interacting with blasts. IL-10 did not impair CART cytotoxicity against B-cell leukemia targets NALM6 or UOCB, and it enhanced the cytolytic capacity of CART22 against NALM6 cells (figure 3A,B). Correspondingly, IL-10 consistently increased target-cell dependent IFNγ secretion from CART cells (figure 3C).

Both enhanced T-cell activation and delayed target cell death can drive increased IFNγ secretion at the level of individual CART/target cell ISs.^18 19^ Further, prolongation of the IS with increased IFNγ secretion is associated with hyperinflammatory states such as hemophagocytic lymphoid histiocytosis.^19^ We hypothesized that IL-10 was increasing IFNγ by prolonging the CART IS. We first compared CART19 and CART22 IS and observed that the CART22 IS duration was significantly longer than the CART19 IS against NALM6 targets (figure 3D). However, the addition of IL-10 affected the CART19 and CART22 IS differently, prolonging the CART19 IS but shortening CART22 IS duration. We observed the same trends with UOCB cells (online supplemental figure 8A).

To identify the transcriptional impact of IL-10, we compared resting CART19 and CART22 cells with and without IL-10 added (online supplemental figure 8B,C). 457 genes were differentially expressed in CART22 cells treated with IL10 compared with those without (online supplemental data 2) versus 48 genes were differentially expressed in CART19 cells treated with IL10 compared with those without (online supplemental data 3). Of these, 21 genes were commonly differentially expressed between these two conditions, with *SOCS3* the most highly expressed (online supplemental figure 8C). To better understand how IL-10 may be influencing CART cells when they interact with target cells, we performed bulk RNA-seq after co-culturing NALM6 cells with CART19 (figure 3E) or CART22 (figure 3F) with or without IL-10 added and found that *SOCS3*, *IL22,* and *CXCL8* were upregulated by IL-10 in both conditions. We found similar (although not statistically significant) results when CART were co-cultured with UOCB cells (online supplemental figure 8D,E). SOCS3 and IL-22 were not measured in the serum; however, CXCL8 (IL-8) levels mirrored IL-10 in patient serum samples, with higher levels in CART22 patients than in CART19 patients with minimal CRS (online supplemental figure 8F).

### Pretreatment peripheral blood cellular populations are transcriptionally distinct in CART22 versus CART19 Patients

**Figure 4:**
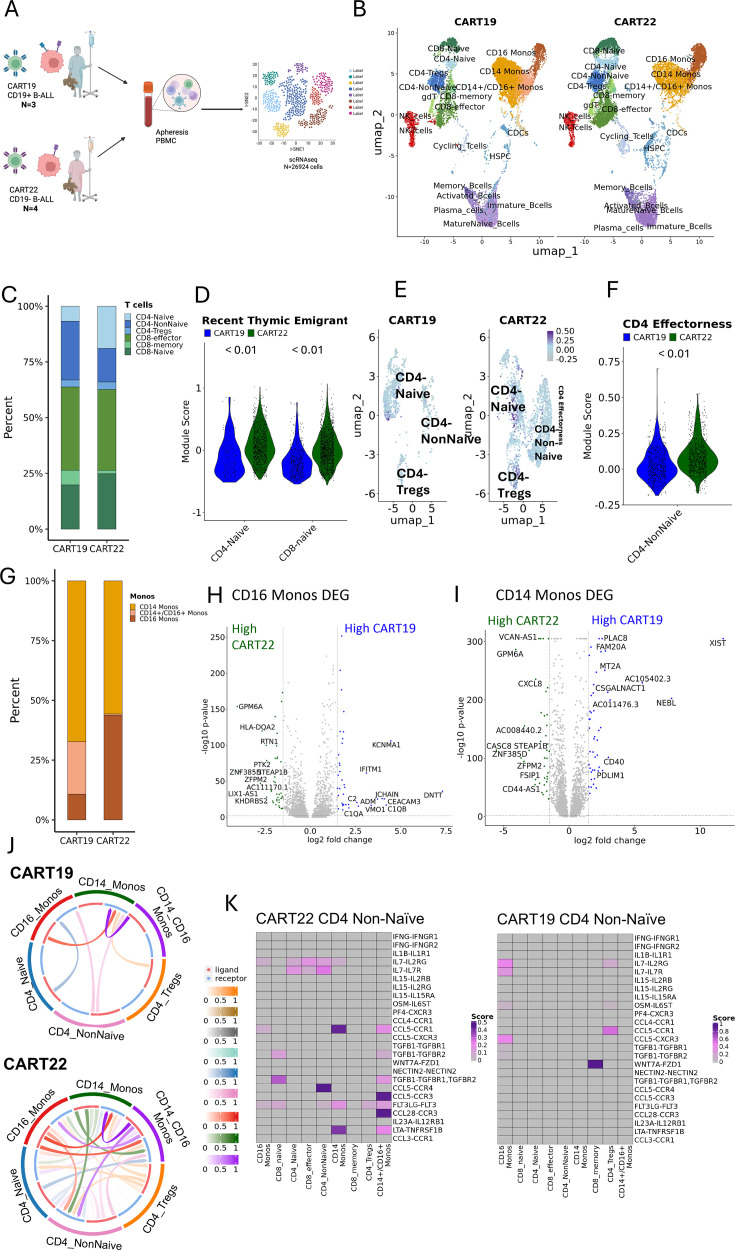
scRNAseq comparing preinfusion PBMCs between patients who went on to receive CART19 and patients who went on to receive CART22. (**A**) Schematic demonstrating experimental design. (**B**) PBMCs at the time of apheresis in CART19 (n=3, n=13,387 cells) and CART22 (n=4, n=13,537 cells) patients were sequenced using scRNAseq. UMAP demonstrating annotations of cell types in CART19 and CART22. (**C**) Proportion of T-cells of each population comparing CART19 and CART22 patients. (**D**) Module score for recent thymic emigrant related genes (*CD38, SOX4, CD7, IKZF2, TOX, IL2RA*) applied to naive T-cell subtypes between CART19 and CART22 patients. P value calculated by Wilcoxon test. CD4 populations had higher effectorness scores across all subtypes as demonstrated by the (**E**) feature plot (purple represents module score) and specifically in non-naïve cells as shown in the (**F**) violin plot CART19 patients colored in blue, CART22 patients colored in green. P values were calculated by Wilcoxon test. (**G**) Monocyte population proportions differed between CART19 and CART22 patients with a higher proportion of CD16 monocytes in CART22 patients compared with CD14 monocytes. DEGs between CART19 and CART22 patients in CD16 (**H**) and CD14 (**I**) monocytes. Log fold change ≥2, adj. p value <0.01. Multiple testing correction was done using the Bonferroni method. Circos plots demonstrating CellChat predicted interactions between monocytes and CD4 T-cells (**J**) in CART19 and CART22 patients. Inner blue arc represents receptor, red arc represents ligand. (**K**) Scoring of CD4 non-naïve T-cells and interacting cells based on specific ligand/receptor pairs by CART22 and CART19 patients, purple represents score. B-ALL, B-cell acute lymphoblastic leukemia; CART, chimeric antigen receptor T-cell; CART19, CD19-directed CART; CART22, CD22-directed CART; DEG, differentially expressed gene; HSPC, Hematopoietic Stem and Progenitor Cells; NK, natural killer; PBMC, peripheral blood mononuclear cell; scRNAseq, single-cell RNA-sequencing; t-SNE, t-distributed stochastic neighbor embedding; UMAP, uniform manifold approximation and projection.

To understand how preinfusion cellular populations differed between the two cohorts, and to identify the source of IL-10 we examined PBMCs from the time of apheresis using scRNAseq (CART19 n=3, CART22 n=4; figure 4A). There were no statistically significant differences between major cell type proportions in CART22 versus CART19 patients (online supplemental figure 9 and online supplemental data 4). A small population of CD34+ Hematopoietic Stem and Progenitor Cells (HSPCs) were seen in both patients, potentially due to recent chemotherapy.

Multiple studies have found that CD4 T-cells and monocytes are the primary cell types implicated in the pathophysiology of CRS.^2 23^ A larger proportion of CD4 T-cells were naïve in the CART22 group than in the CART19 group (8.1% vs 1.2% respectively, $\chi^{2}$<0.001, figure 4C). Correspondingly, a recent thymic emigrant gene module was elevated in naïve populations in the CART22 relative to the CART19 group (figure 4D; online supplemental data 5).^24^ To investigate the relative activation state of non-naïve CD4 T-cells, we applied a recently described CD4 T-cell “effectorness” score to further characterize differences between CART22 and CART19 patients.^25^ We found higher effectorness scores in the CART22 than CART19 patients in non-naïve CD4 T-cells (figure 4E,F).^25^ As a validation of this result, we applied the Azimuth CD4 effectorness score with a similar result (online supplemental figure 10). In summary, patients who went on to receive CART22 had a higher proportion of naïve CD4 T-cells, and evidence of great potential for effectorness in non-naïve populations.

Monocyte populations also differed with a higher proportion of CD16+ monocytes in CART22 patients than CART19 patients (figure 4G, 43.8% vs 11.0% of total monocytes, $\chi^{2}$ <0.001). CD16+ (non-classical) monocytes are associated with a higher inflammatory potential.^26^ When comparing CD16+ monocytes 341 differentially expressed genes (DEGs) were significantly differentially expressed between CART19 and CART22 patients, with 282 DEGs in CD14+ monocytes (figure 4H,I**,** online supplemental data 6 and 7). Notably, CXCL8 was more highly expressed in CART22 CD14+ monocytes than in CART19 CD14+ monocytes.

Using CellChat, a computational tool that uses scRNAseq to predict cell-cell interactions and quantitively infer interaction strength, we investigated interactions between CD4 T-cells and monocytes (figure 4J).^27^ In keeping with the finding of higher CD4 T-cell “effectorness”, CART22 patients had more and stronger predicted interactions involving monocytes than CART19 patients. We specifically examined non-naïve CD4 T-cells and found that IL7, IL2RG/IL7R and CCL5-mediated interactions were more characteristic of interactions in the CART22 than the CART19 cohort (figure 4K).^23^ Finally, we examined *IL10* expression levels and identified minimal expression across cell types (online supplemental figure 11).

### The bone marrow microenvironment differs between CART19 and CART22 patients

**Figure 5:**
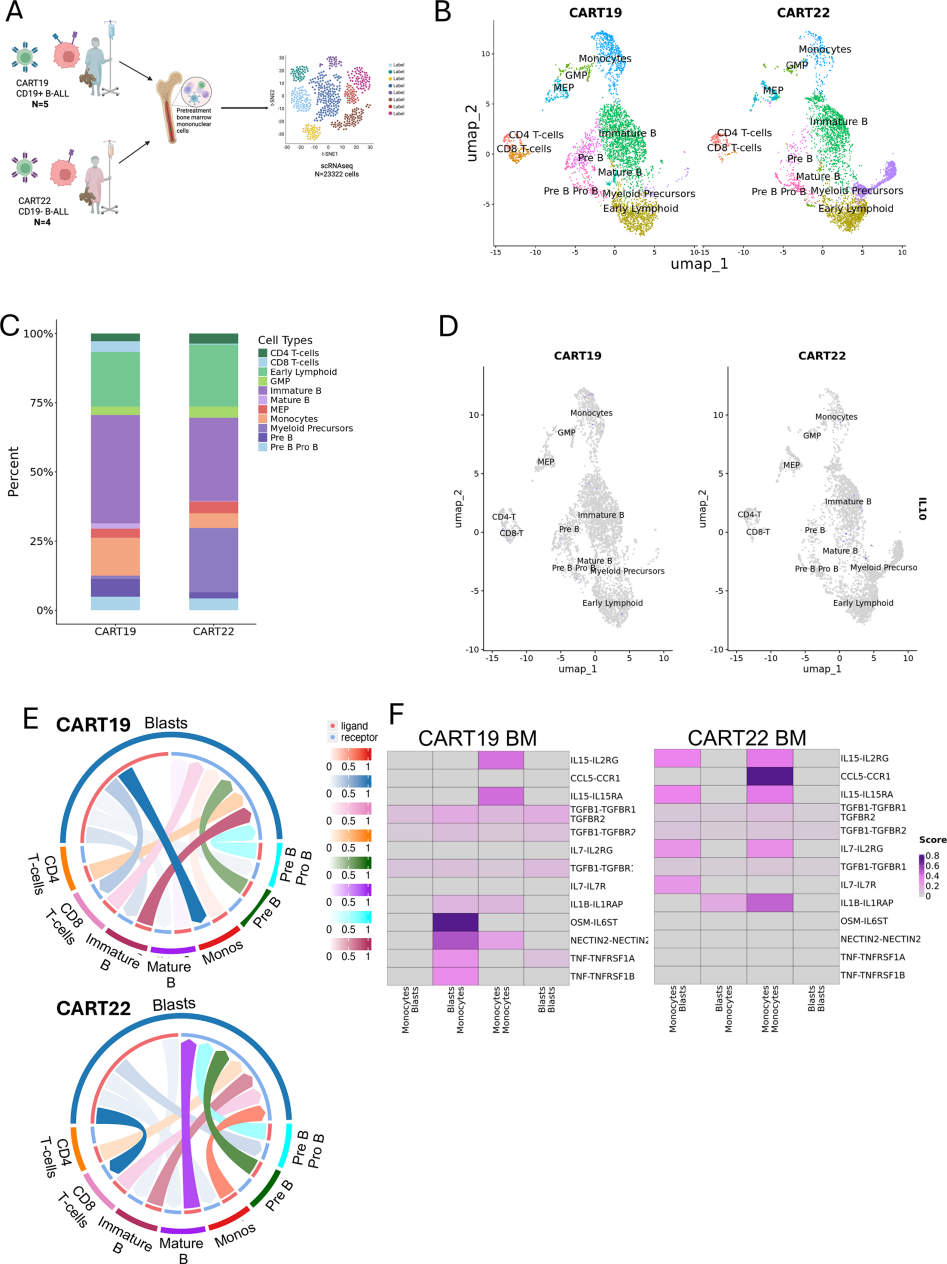
scRNAseq comparing preinfusion bone marrow between patients who went on to receive CART19 and patients who went on to receive CART22. (**A**) Preinfusion BMMCs in CART19 (n=4,330 cells, n=5 patients) and CART22 (n=4,333 cells, n=4 patients) patients were sequenced using scRNAseq. (**B**) UMAP demonstrating annotations of BMMCs of CART19 and CART22. (**C**) Proportion of each cell population comparing CART19 and CART22 patients. (**D**) Feature plot showing no expression of IL10 in any cell population in CART19 and CART22 patients. (**E**) CellChat cell–cell interaction analyses was completed. Circos plots demonstrating predicted interactions and predicted interaction strengths between blasts and other marrow cell types in CART19 and CART22 patients. Inner blue arc represents receptor, red arc represents ligand. (**F**) CellChat predicted interaction strength score of interactions between blasts and monocytes in CART22 and CART19 patients based on CellChat ligand/receptor pairs database. B-ALL, B-cell acute lymphoblastic leukemia; BMMCs, bone marrow mononuclear cells; CART, chimeric antigen receptor T-cell; CART19, CD19-directed CART; CART22, CD22-directed CART; IL, interleukin; scRNAseq, single-cell RNA-sequencing; t-SNE, t-distributed stochastic neighbor embedding; UMAP, uniform manifold approximation and projection.

To investigate differences in the bone marrow microenvironment between CART19 and CART22 treated patients, we performed scRNAseq on BMMCs from preinfusion bone marrow aspirate from a cohort of five CART19 (n=14,903) and four CART22 (n=8,419 cells) patients (figure 5A). Blasts were removed for annotation (n=1,237 and 4,075, CART19 vs CART22; online supplemental data 8). For visualization purposes the CART19 marrow object was downsampled randomly, with the full cohort used for statistical comparisons (figure 5B). There was a higher proportion of myeloid precursors in CART22 patients compared with CART19 patients; no other cell types had statistically significant differences (figure 5C**;** online supplemental figure 12 and online supplemental data 9). There was no apparent difference in *IL10* expression between CART19 and CART22 BMMCs, with minimal expression overall (figure 5D).

We hypothesized that CD19-negative blasts might interact differently with the marrow microenvironment than CD19-positive blasts. 577 genes were differentially expressed between CD19 positive and CD19 negative blasts. As expected, CD19 expression was higher in the CD19-positive blasts (online supplemental data 10). *IFNLR1*, which encodes a receptor that dimerizes with IL10RB to form a Type III IFN receptor, was more highly expressed in blasts from CART22 patients compared with CART19 patients (online supplemental data 10).^28^ Predicted cell–cell interactions demonstrated overall more interactions between blasts and other cell types in the CART22 patients than in the CART19 patients (figure 5E). These receptor/ligand differences appeared to be driven by strong TNF/TNFRSF1A/B, NECTIN2, and OSM (oncostatin)/IL6ST in the CART19 patients and IL7/IL15 interactions in the CART22 patients (figure 5F).

### Evidence of higher IL-10 exposure in cellular populations of Patients who went on to receive CART22

**Figure 6:**
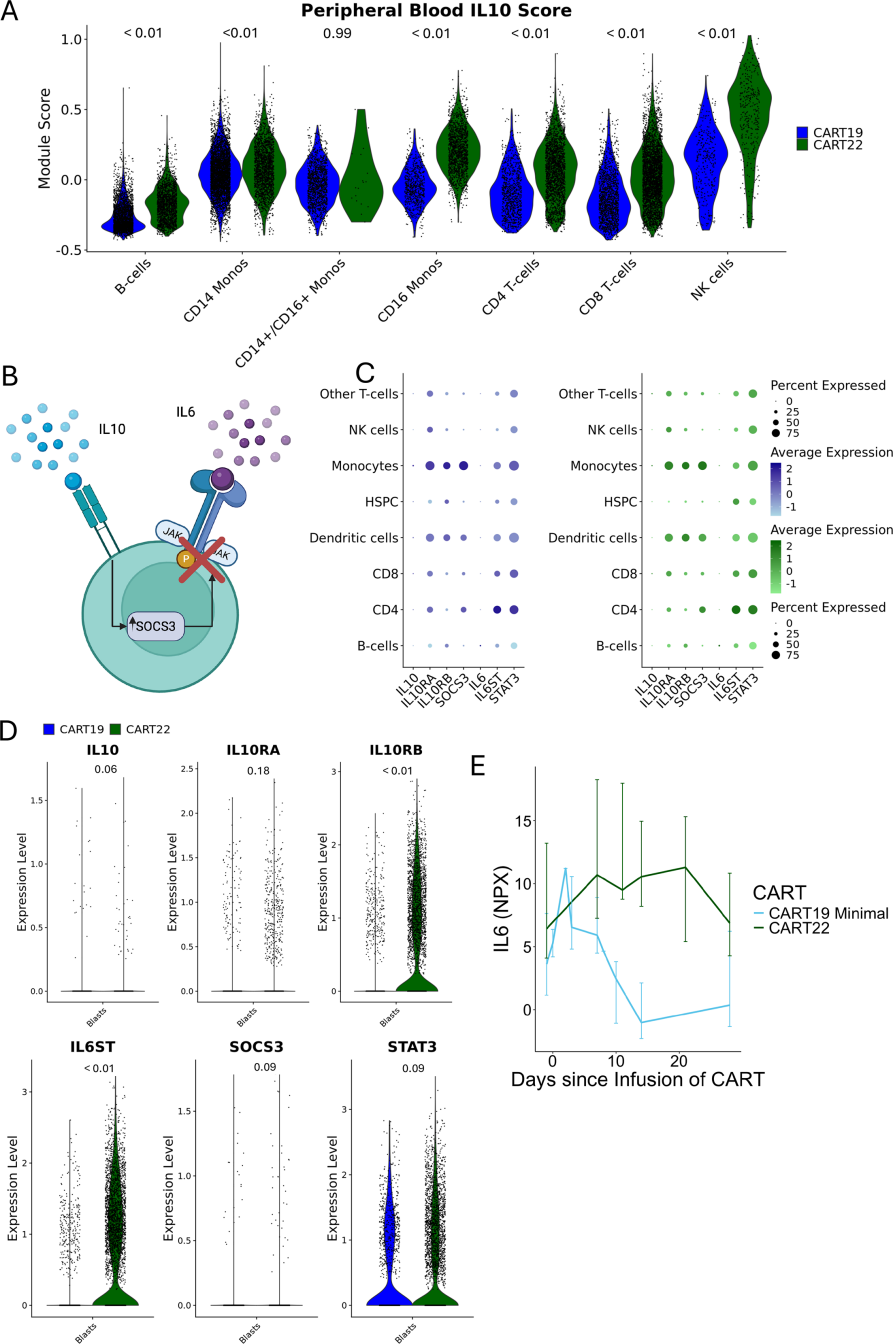
scRNAseq reveals evidence of IL-10 effect on PBMCs cellular populations. (**A**) Violin plot of “IL-10 score” in scRNAseq of PBMC populations between CART19 (blue) and CART22 (green) patients. P value calculated with Wilcoxon test (**B**) Schematic representation of IL-10/SOCS3/IL-6 relationship in CD4 T-cells and other cellular populations. (**C**) Dot plots demonstrating *IL10* and associated gene expression in different cell types between CART19 (blue) and CART22 (green) patients. (**D**) Expression of *IL-10* and related genes in blasts from patients who went on to receive CART19 compared with those who went on to receive CART22. All p values were calculated using Wilcoxon test. (**E**) Serum protein measurement of median IL-6 (NPX) comparing CART22 patients (n=17) to patients with CART19 minimal CRS (n=13). Error bars represent IQR. CART, chimeric antigen receptor T-cell; CART19, CD19-directed CART; CART22, CD22-directed CART; CRS, cytokine release syndrome; HSPC, Hematopoietic Stem and Progenitor Cells; IL, interleukin; NK, natural killer; NPX, normalized protein expression; PBMCs, peripheral blood mononuclear cells; scRNAseq, single-cell RNA-sequencing.

We next developed an “IL-10 Response Score” based on the 21 genes commonly upregulated in our in vitro IL-10 experiment (online supplemental figure 4C and online supplemental data 11). As these genes were specific to T-cells, we cross-referenced this list with human genes found to be upregulated in monocytes when mice were injected with IL-10 (Cytokine Dictionary).^29^ We included eight genes that were upregulated in at least 2 of 3 conditions when IL-10 was added, with a log fold-change of at least 2 (online supplemental data 12). A total of eight genes overlapped across the lists, including *SOCS3, ADD2, BCL3, EGFL7 (NEU1), GZMB, IFITM1, IFITM2,* and *SH3RF3*. We validated these genes in an aggregator of data obtained from cytokine-based experiments (CytoSig) and 7 of 8 (all except for *ADD2*) had been previously reported to be associated with IL-10 in experimental data.^30^ We used the seven genes for our final “IL-10 response score”. Higher IL-10 responsiveness scores were present across all cell types in CART22 patients compared with CART19 patients (figure 6A PBMCs and online supplemental figure 13 BMMCs). The difference was especially marked and statistically significant in CD4 T-cells, CD8 T-cells, monocytes and natural killer cells. When *SOCS3* (the most highly DEG) alone was examined, expression was higher in CART22 patients in CD4 T-cells and CD16+ monocytes, and higher in CART19 patients in CD14+ monocytes (online supplemental figure 14). SOCS3 functions as a negative regulator of responsiveness to IL-6 family cytokines by binding to phosphorylated gp130 (IL6ST) and inhibiting downstream signaling (figure 6B).^31 32^ Though IL-10 signals via STAT3, it does not use gp130 and its signaling is therefore unaffected by SOCS3.^31^ We examined genes associated with this pathway (*IL10, IL10RA, IL10RB, SOCS3, IL6, IL6ST, STAT3*) by cell type (figure 6C). Activation of this pathway was most obvious in CD4 T-cells, CD8 T-cells, dendritic cells, and monocytes. *IL10RA* levels were significantly higher in CART19 compared with CART22 patients across multiple cell types (figure 6D and online supplemental figure 15), in keeping with serum protein measurement of IL10-R1 (the protein encoded by *IL10RA*, online supplemental figure 6). We hypothesized that higher soluble IL10-R1 in CART19 patients may be acting as a “sink” for free IL-10, leading to lower levels of circulating IL-10 in CART19 patients. When we examined the same genes in blasts, we noted higher expression in CART22 blasts compared with CART19 blasts, particularly for *IL6ST, IL10RB* and *STAT3* (figure 6E).

We hypothesized that in the presence of negative regulation of IL-6 via SOCS3, there would be compensatory excessive production of IL-6. Levels of serum IL-6 are impacted by tocilizumab, and all patients in the severe CRS cohort received tocilizumab. We therefore compared IL-6 levels over time between CART22 patients and patients with CART19 minimal CRS (figure 6F). As expected, we saw much higher IL-6 levels in CART22 patients than in CART19 patients with minimal CRS, despite no clinical severe CRS in CART22 patients.

## Discussion

Our work demonstrates that higher endogenous IL-10 levels prior to treatment with CAR-T correlates with milder CRS in patients, and importantly, that the addition of IL-10 had a differential impact on CART19 and CART22 cells with preserved antileukemic efficacy. Furthermore, we observed that in patients with high disease burden CART22 induced a distinct immune response with elevated IL-10 activity, attenuated CRS and preserved proliferation compared with CART19.

We observed striking differences between IL-10 levels in the preinfusion serum of patients who went on to receive CART22 (all of whom had previously received CART19) and those who went on to receive CART19 (and were CART-naïve). In patients who went on to receive CART22, IL10 levels tended to correlate with disease burden. We validated this finding in a separate cohort of patients and found that IL-10 levels were increased in patients who had previously been treated with CART19 and had measurable residual disease by flow cytometry, and that in this cohort (but not in the CART-naïve cohort) IL-10 levels correlated with disease burden. This implies that IL-10 is the result of an interaction between relapsed leukemia and the broader immune microenvironment. The exact mechanism of this phenomenon requires further study; nevertheless, this demonstrates that immunotherapy has the potential to permanently alter the immune microenvironment. Further work should examine the impact of prior receipt of other immunotherapies, including blinatumomab, on the immune microenvironment in the setting of relapsed leukemia.

IL-10 is a pleiotropic cytokine with multiple, context-specific physiologic roles. We demonstrate a differential impact of exogenous IL-10 on CART-blast interactions at the level of the IS. Both CART used contained a 4–1BB costimulatory domain with almost identical structures except for the single-chain variable fragment. The CART22 product contains a short linker which has been previously demonstrated to have a higher level of antigen-independent activation.^16^ In addition to these known differences, IL-10 induced different gene expression profiles when applied to CART19 or CART22 cells alone. This construct-specific effects on the CART-blast IS has not been previously reported and implies a key regulatory role of IL-10 in CART-blast interactions.

In our in vitro experiments, we demonstrate that IL-10 increases the suppressor of cytokine signaling protein SOCS3 in CART cells. This finding was corroborated by elevations of *SOCS3* in our transcriptional data. SOCS3 is a specific inhibitor of IL-6 effect via its ability to specifically bind gp130 and inhibit STAT-mediated signaling of receptor-bound IL-6.^32 33^ This leads to high specificity for receptor-bound IL-6, and makes SOCS3 an ideal target for potential therapeutic exploitation. Intracellular SOCS3 modulation has previously been demonstrated to suppress acute inflammation.^34^ In patients at high risk for severe CRS, either direct, transient delivery of SOCS3 protein or upregulation of SOCS3 via preinfusion IL-10 stimulation or genetic manipulation could potentially abrogate the impact of CRS.

Our study has several limitations. First, although we have serum protein data on a cohort of 40 patients, our single cell analyses are limited by a small number of patients on whom residual samples are available, and these observations require further validation in subsequent studies. Although we measured significant increases in serum IL-10 levels in both peripheral blood and marrow serum and clearly demonstrated the effects of IL-10 on cellular populations in our scRNAseq, we were unable to determine the source of IL-10 from our PBMC or BMMC data. This may be due to poor RNA-protein expression correlation of ILs, or alternatively, IL-10 production may be primarily from a cell type that was not measured, such as tissue-resident macrophages, marrow stromal elements or endothelial cells.^35^ Further work is required to conclusively determine if IL-10 is modulating the severity of CRS. All patients examined in this study were treated with 4–1BB containing CART, and these effects may differ in patients treated with CART of different designs, or in patients who are less heavily pretreated. Finally, although no severe CRS occurred in our cohort of patients treated with CART22, other groups have reported the occurrence of cases of severe CRS in patients treated with CART22.^36^ Future work should specifically examine cytokine and cellular features in these patients to determine the factors associated with severe CRS, and how severe CRS following CART22 differs from severe CRS following CART19.

In summary, our work identifies IL-10 as a potential central modulator of the immune storm triggered by CART. We demonstrate that in the setting of relapsed leukemia, previous immunotherapy may alter serum IL-10 levels. Given the expanding use of CART, strategies to fine-tune immune activation are increasingly important. Our findings lay the groundwork for a new generation of toxicity mitigation strategies, potentially expanding the therapeutic window for CART.

## Supplementary material

10.1136/jitc-2026-015126 online supplemental file 1

10.1136/jitc-2026-015126 online supplemental file 2

10.1136/jitc-2026-015126 online supplemental file 3

## Data Availability

Data are available in a public, open access repository.
