# Supplementary material for: Differential cytokine architecture in patients treated with CART19 versus CART22
Source: J Immunother Cancer. 2026 Jun 19;14(6):e015126. doi: 10.1136/jitc-2026-015126 (PMC13289296; doi:10.1136/jitc-2026-015126)
Supplement: online supplemental file 1 [file jitc-14-6-s001.docx]

**Supplemental Methods**

*Quantitative Polymerase Chain Reaction (qPCR)*

qPCR was performed with ABI TaqMan technology targeting specific sequences of the integrated transgene, as previously described.^14^ Each assay was conducted in triplicate using 200 ng of genomic DNA. The number of transgene copies per microgram of DNA was calculated using the formula: Transgene copies per μg of DNA = [(Copies from the standard curve × Correction factor) ÷ DNA input in ng] × 1000.

*Immune Synapse Experiments*

1×10⁵ NALM6 cells were seeded onto 30 mm × 10 mm tissue culture dishes with 15 mm glass bottoms (CellTreat) pre-coated with poly-L-lysine. NALM6 cells were added and incubated overnight at 37°C in 5% CO_2_.

Recombinant human IL-10 (Thermofisher, Cat# 200-10-100UG) was added 10 minutes prior to imaging at a concentration of 10 ng/mL. Immediately prior to imaging, Propidium Iodide (PI) was added to cultures at a final concentration of 5 μM, and CART T cells were labelled with Fluo-4 AM (1 μM Fluo-4 and 0.02% [w/v] Pluronic F-127) for 20 minutes at 37°C before being added to the co-cultures.

Imaging was conducted on a DMi8 confocal microscope (Leica) equipped with a 40× (NA 0.85) air objective and LAS AF software (Leica). Sequential optical sections were acquired through the center of the cells using the following channels: Fluo-4 (excitation 488 nm), PI (excitation 561 nm), and brightfield/differential interference contrast. The pinhole for the 488 and 561 channels was set to 4.2 AU, yielding an optical section thickness of 5 µm and an XY pixel size of 378.8 nm. Images were captured at a rate of 10 frames per minute.

*Cytokine Measurements*

Cytokine production was assessed from 50 μL of supernatant collected from co-cultures of CART and target cells (NALM6 or UOCB) at a 1:3 effector-to-target (E:T) ratio. Interferon gamma (IFNγ) levels were quantified using Cytometric Bead Array (CBA) Flex Sets (BD Biosciences), following the manufacturer’s instructions. Samples were analyzed using a CytoFLEX5 flow cytometer (Beckman Coulter).

Cytokine concentrations were measured using a custom 32‑plex human panel (EMD Millipore Corporation), which included IL‑10. Each sample was assayed in duplicate following the manufacturer’s instructions and quantified against multiple internal standards with a six‑point calibration curve. Data were acquired on a FlexMAP‑3D system (Luminex) and analyzed using XPonent 4.3 software (Luminex). Values falling below the assay’s detection limit were imputed according to the formula (minimum standard value × dilution factor) / 2.

*Single Cell RNA Sequencing (scRNA-seq)*

Cryopreserved peripheral blood mononuclear cells (PBMCs) or bone marrow mononuclear cells (BMMC) were thawed and resuspended in media. Suspensions were centrifuged at 300 x g for 5 minutes at 4°C and resuspended in Dulbecco's phosphate-buffered saline (DPBS, Gibco, PN 14190-144) with 1% bovine serum albumin (BSA, Miltenyi Biotec, PN 130-091-376). Cell suspensions were then centrifuged at 500 x g for 5 minutes at 4°C and resuspended in 50 µL of cell staining buffer (Biolegend Inc, PN 420201) followed by addition of 5 µL of Human TruStain FcX™ blocking reagent (Biolegend Inc, PN 422301) if the cell count per sample in DPBS + 1% BSA was > 200,000 cells. Cells were incubated in Fc block for 10 minutes at 4°C. During incubation, hashtag antibodies were prepared by adding 2.2 µL (1.5 µg) of each of the TotalSeq™ anti-human Hashtag to 52.8 µL cell staining buffer and centrifuged at 14000 x g for 10 minutes at 4°C. At the end of incubation in Fc block, each cell suspension was labelled with 50 µL TotalSeq™ anti-human Hashtag antibody and incubated for 30 minutes at 4°C. Cells were then washed thrice in cell staining buffer at 500 x g for 5 minutes at 4°C and resuspended in DPBS + 0.04% BSA. If cell counts in DPBS + 1% BSA were < 200, 000 cells, cells were centrifuged at 300 x g for 5 minutes at 4°C and resuspended in DPBS + 0.04% BSA. Hashed and pooled cell suspensions or individual cell suspensions were subsequently processed to generate single cell RNA-seq libraries using chromium Next GEM single cell 3’ reagent kits v3.1 Dual Index (10x Genomics, PN-1000268) with 3’ feature barcode technology (10x Genomics, PN-1000262) for cell surface protein using chromium controller as per manufacturer’s instructions. Library fragment size was determined using the Bioanalyzer Agilent 2100 with the High Sensitivity DNA chip (Agilent Technologies, 5067-4626) and libraries were quantified using Kapa qPCR quantification kit (Roche Diagnostics, PN 501965234). After pooling, libraries were sequenced on an Illumina NovaSeq 6000 using sequencing parameters read1: i7: i5: read2 :: 28:10:10:90 bp at an average sequencing depth of 50,000 read pairs per cell.

Olink Data Analysis

Eight bridging samples were included with CART22 samples to bridge the CART19 and CART22 data sets. Normalization was completed as recommended by Olink®. The median difference of each protein within the bridging samples was calculated. The protein-specific median was then added to each protein value to normalize the two datasets.

P-values were calculated using as the false discovery rate (FDR) with a cutoff of 0.05 and a fold-change cutoff of 2. All analyses were performed in R using ggpubr (version 0.6.0), pheatmaps (version 1.0.12), eulerr (version 7.0.2) and base R.

Bulk RNASeq Data Analysis

Fastq files were assessed for quality control using the FastQC program and aligned to the human reference genome (GRCh38) using the STAR aligner (v2.7.11b). Duplicate reads were flagged using the MarkDuplicates program in Picard tools. Per gene read counts for Ensembl gene annotations were computed using the subread package in R (v 2.18.0), and duplicate reads were removed. Gene counts were normalized for library sizes and represented as counts per million (CPM) using the edgeR R package (v 4.2.2). Genes with a CPM < 1 in 25% of samples were removed as lowly expressed.

Data were transformed using the voom function from the limma package (v 3.60.6) in R. Differential gene expression was performed using a linear model with the limma package. Volcano plots were generated in R using the ggplot2 package (v 3.5.2)

scRNAseq Analysis

Raw data was processed using cell-ranger (version cellranger-8.0.1). Cell ranger matrix files were run through SoupX (version 1.6.2) to remove any cell-free RNA contamination. The SoupX files were loaded into Seurat v5.1.0 for further QC and analyses. Data was filtered based on hemoglobin and mitochondrial gene counts (HBB genes sum < 3 and Mitochondrial percent <= 20% per cell) and single-cell nCounts (40,000 < nCount > 1500). Doublets were further filtered out using DoubletFinder v.2.0.3. Samples in each group were integrated using RPCAIntegration in Seurat. Data was annotated using Azimuth v.0.5.0 and manual annotation of each cell type cluster.

For bone-marrow single-cell samples, down-sampling was performed to allow for comparison between the CART19 and CART22 groups. Down-sampling was done by randomly removing cells from our CART19 group while preserving the proportions of each annotated cell-type within the group.

Blast cells were identified using infercnv v.1.21.0 and using B-ALL blasts gene markers (*DNTT*, *MME*, *CD19*, *MS4A1*, *CD22*). Reference cells were identified in the bone marrow samples by projecting the single-cell data to a healthy bone marrow reference^38^. Cells that projected over healthy reference clusters were labeled as normal cells and fed as a reference to infercnv. To identify cell-to-cell interactions, packages cellcall v. 1.0.7 and CellChat v. 2.1.2 were run. Enrichment scores were calculated using Seurat AddModuleScore function. Pathway analyses were done using packages ReactomePA v.1.46.0 and clusterProfiler v.4.10.1. Single-cell differential expression analyses were done using FindMarkers function in Seurat with a min.pct = 0.2. Proportion testing across cell types was completed using the propeller function in Speckle v.1.2.0. Statistical analyses were completed using the ggpubr v.0.6.0 package.
