# Supplementary material for: Differential cytokine architecture in patients treated with CART19 versus CART22
Source: J Immunother Cancer. 2026 Jun 19;14(6):e015126. doi: 10.1136/jitc-2026-015126 (PMC13289296; doi:10.1136/jitc-2026-015126)
Supplement: online supplemental file 2 [file jitc-14-6-s002.pdf]

Supplemental Table 1. Demographic data.

	CART19 (N=23)	CART22 (N=17)	Overall (N=40)
**Age at infusion, median (range)**	8.9 (1.4, 29.1)	16.1 (3.1, 28.2)	11.3 (1.4, 29.1)
**Female sex, N (%)**	7 (30.4%)	4 (23.5%)	11 (27.5%)
**Trisomy 21, N(%)**	1 (4.3%)	1 (5.9%)	2 (5.0%)
**Number of prior relapses, median (range)**	2 (1,4)	3 (2,4)	3 (1,4)
**Previous CART, N(%)**	0 (0)	16 (94.1)	16 (40)
**Previous HSCT, N(%)**	9 (39.1)	9 (52.9)	18 (45)
**Previous blinatumomab, N(%)**	4 (17.4)	4 (23.5)	8 (20)
**Previous inotuzumab, N(%)**	2 (8.7)	7 (41.2)	9 (22.5)
**CRS Grade, N(%)**			
0	3 (13.0)	3 (17.6)	6 (15)
1	9 (39.1)	9 (52.9)	18 (45)
2	1 (4.3)	5 (29.4)	6 (15)
3	0	0	0
4	10 (43.5)	0	10 (25)

Supplemental Figure 1.

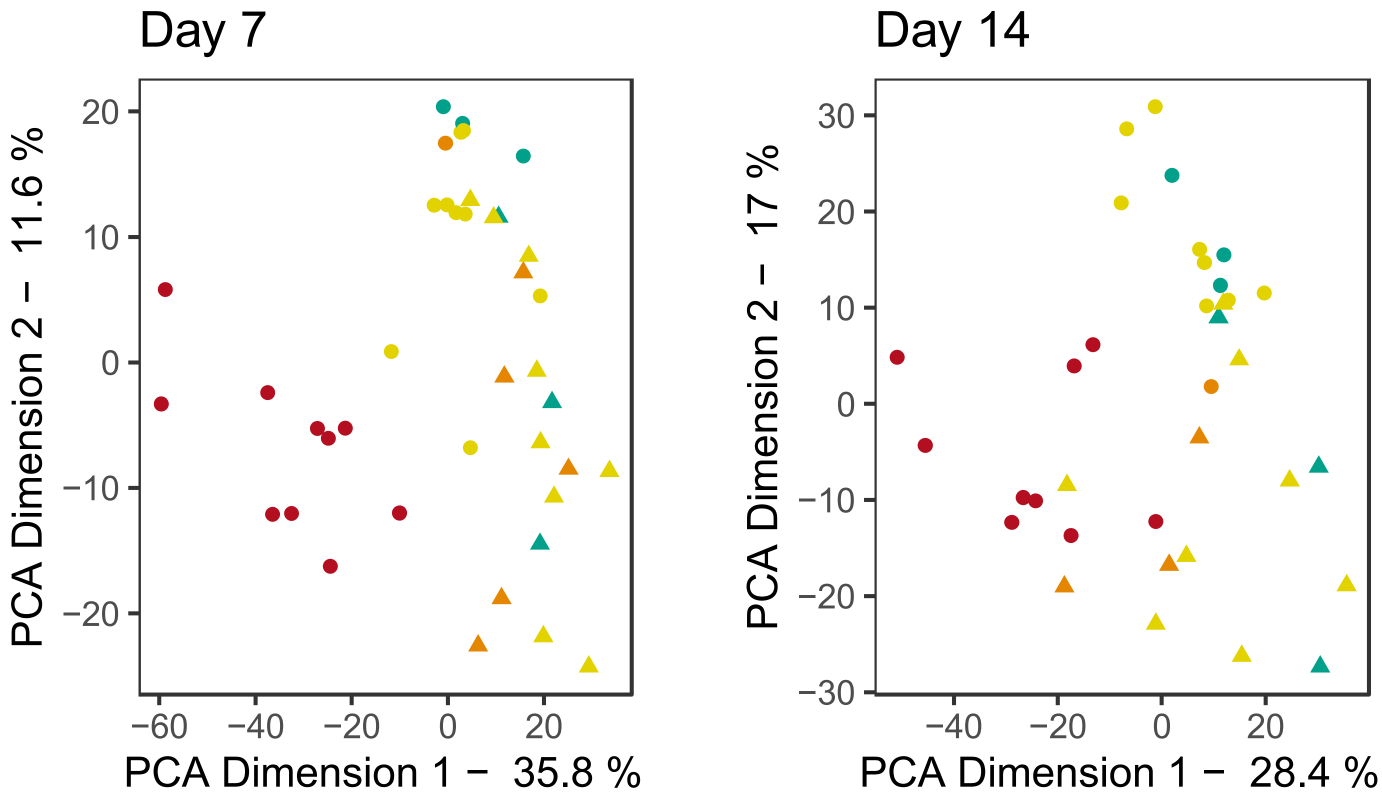

Principal component analysis (PCA) of CART19 (circles) and CART22 (triangles) patients at the Day 7 and Day 14 timepoints.

Supplemental Figure 2.

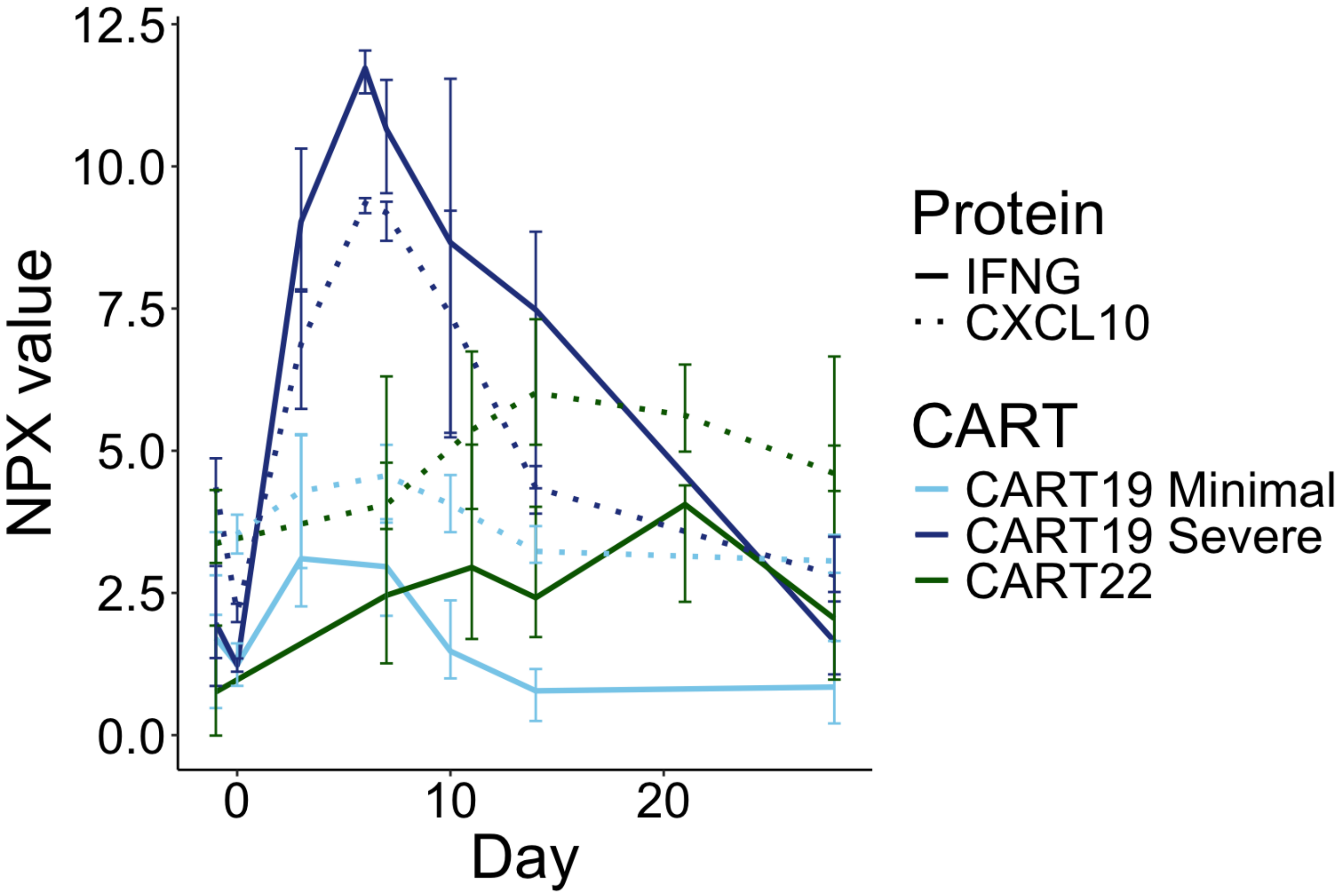

Median serum expression of IFNG (solid line) and the IFN-responsive protein CXCL10 (dotted line) in patients who received CART19 and developed severe CRS (N=10, dark blue) or minimal CRS (N=13, light blue) or who received CART22 (N=17, green). Error bars represent IQR.

Supplemental Figure 3.

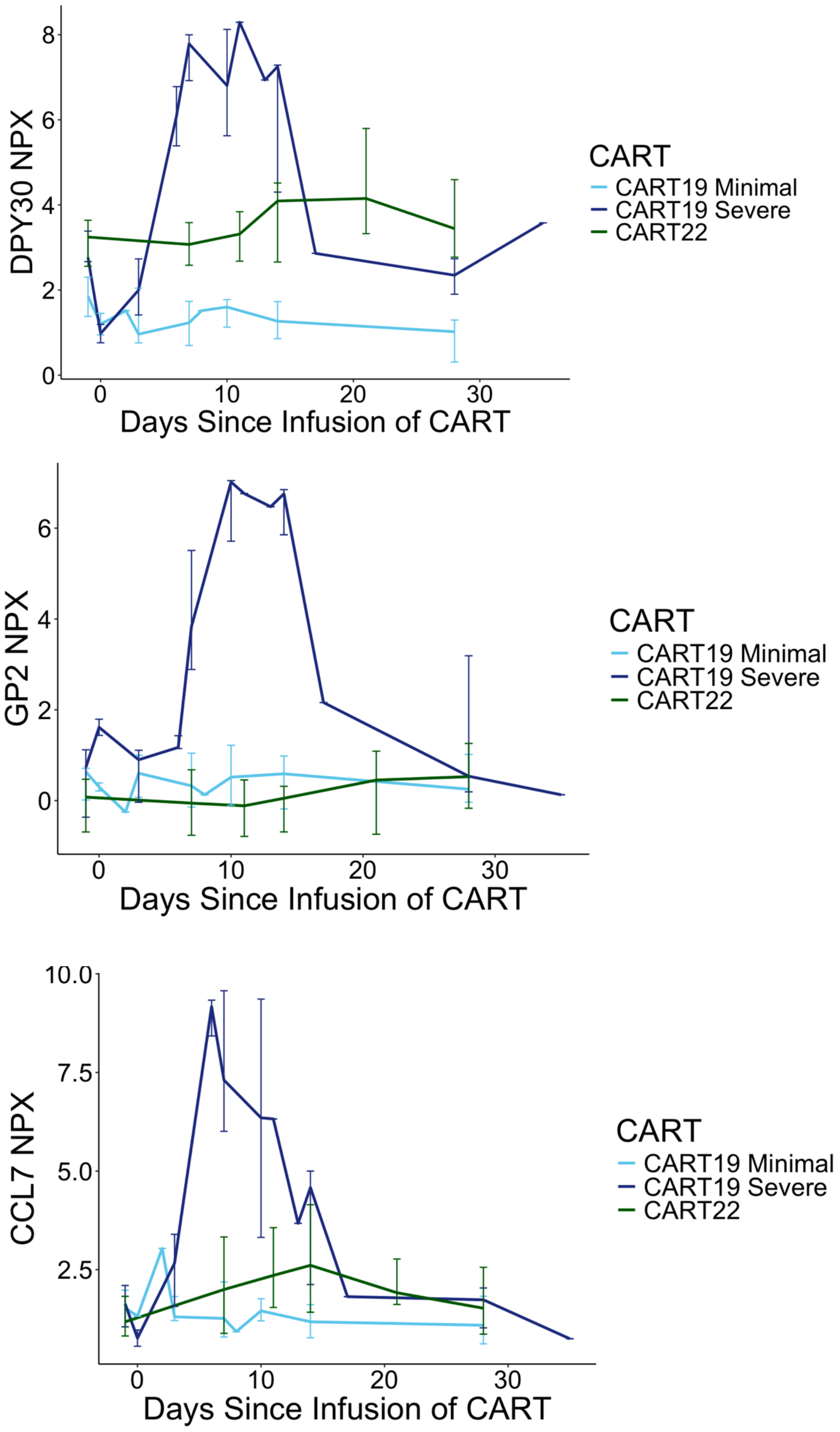

Median serum expression of DPY30, CP2 and CCL7 in patients who received CART19 and developed severe CRS (N=10, dark blue) or minimal CRS (N=13, light blue) or who received CART22 (N=17, green). Error bars represent IQR.

Supplemental Figure 4.

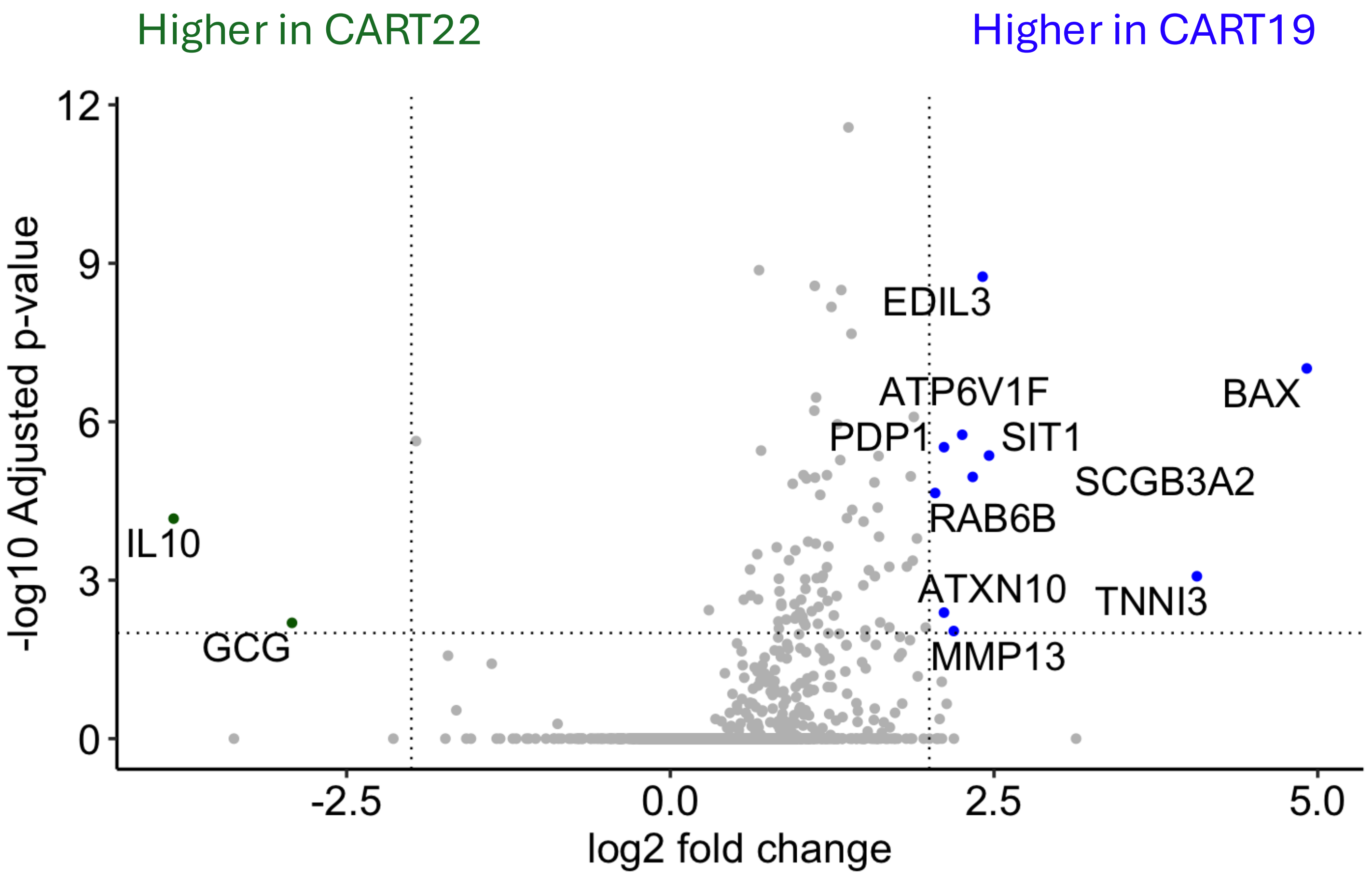

Differentially expressed proteins at the preinfusion timepoint in patients who went on to receive CART22 (N=17) and those who went on to receive CART19 (N=23). Coloured proteins are those with log2 fold change ≥2 and adjusted p-value ≤0.01 (Benjamini-Hochberg). Green is up in CART22, blue is up in CART19.

Supplemental Figure 5.

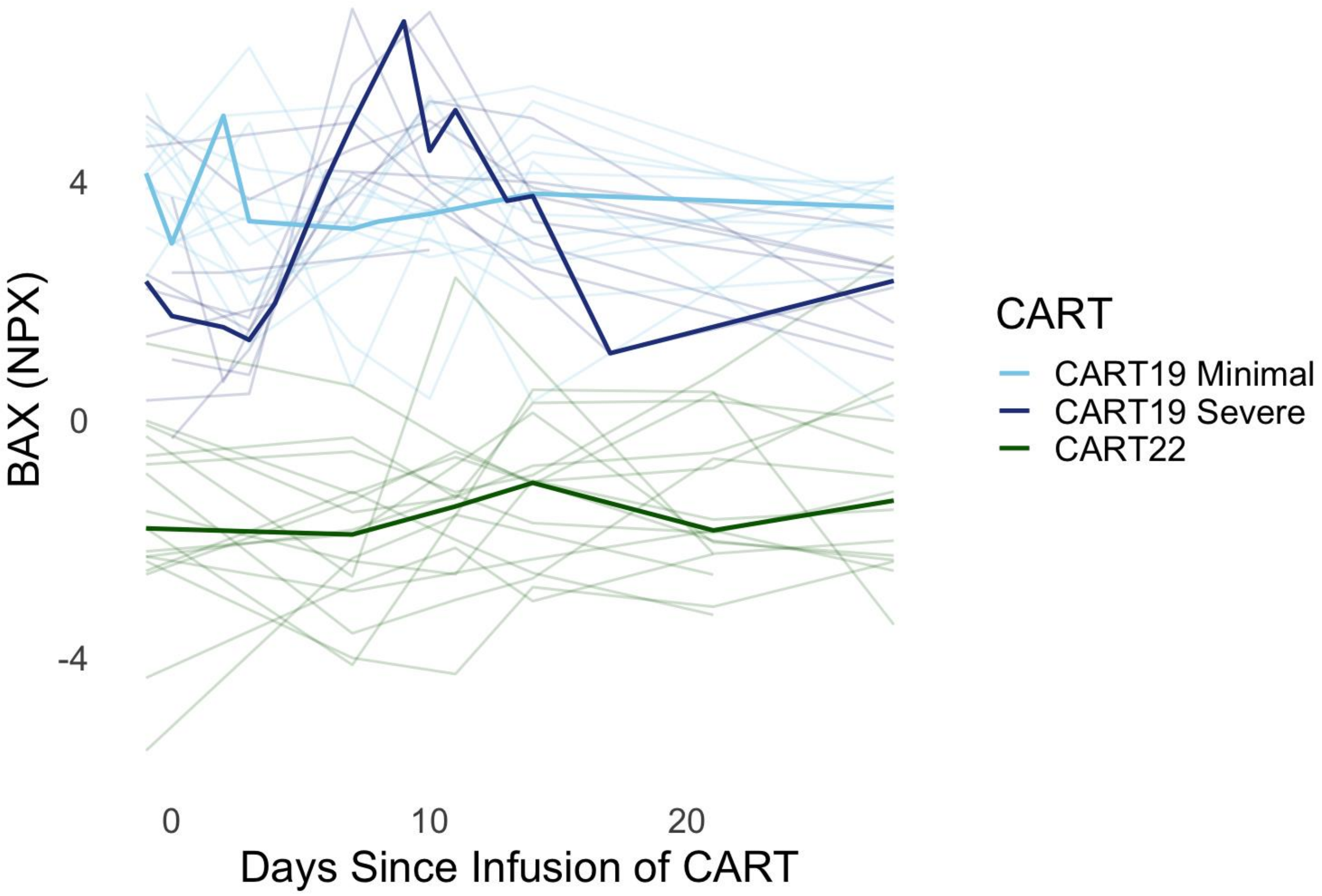

Serum expression of BAX in patients who received CART19 and developed severe CRS (N=10, dark blue) or minimal CRS (N=13, light blue) or who received CART22 (N=17, green). Median is shown in bold lines, each line represent a single patient's data over time.

Supplemental Figure 6.

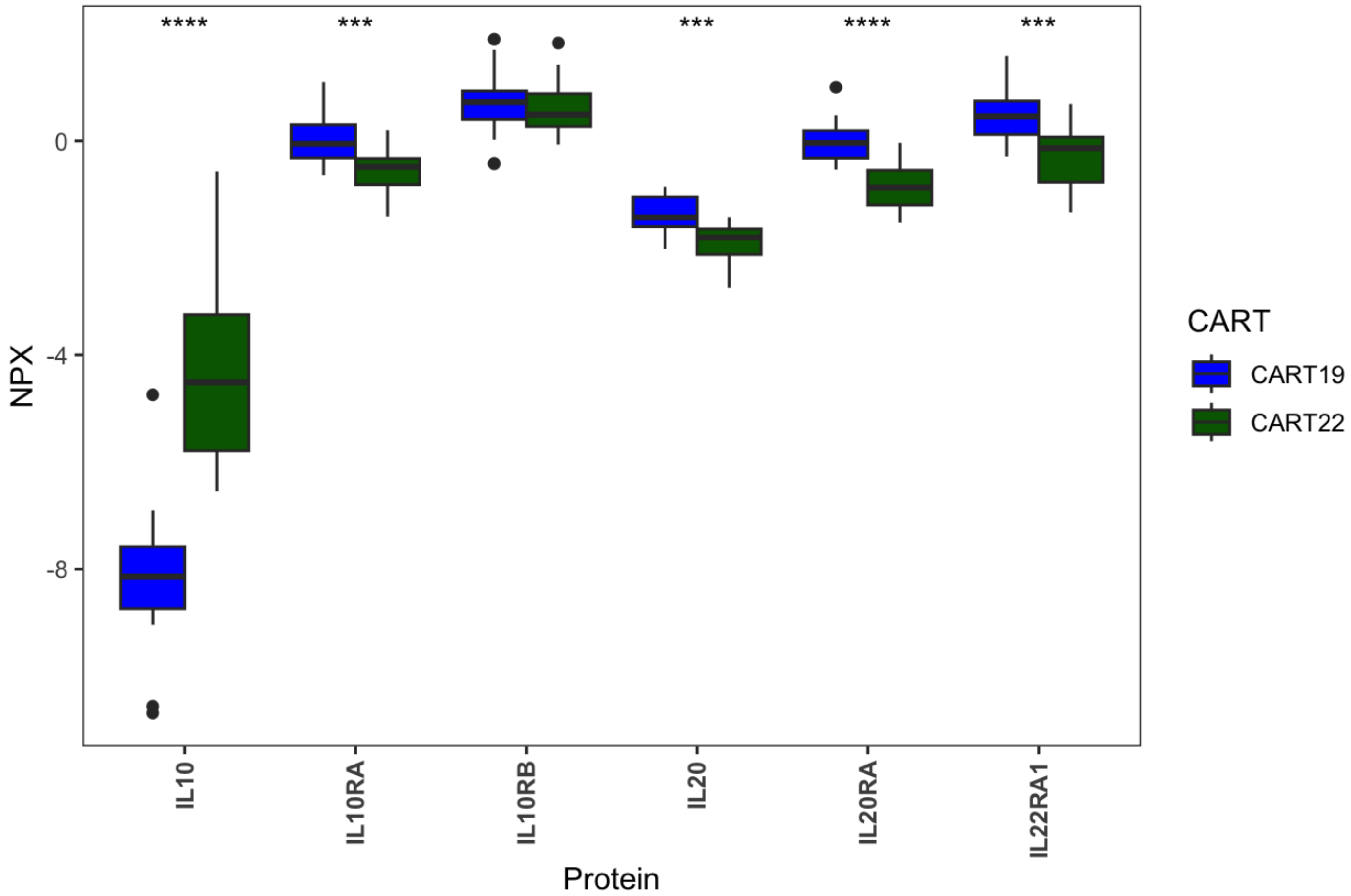

Median serum expression of IL-10 family proteins measured by Olink at the preinfusion timepoint in patients who received CART19 (N=23, blue) or who received CART22 (N=17, green).

Supplemental Figure 7.

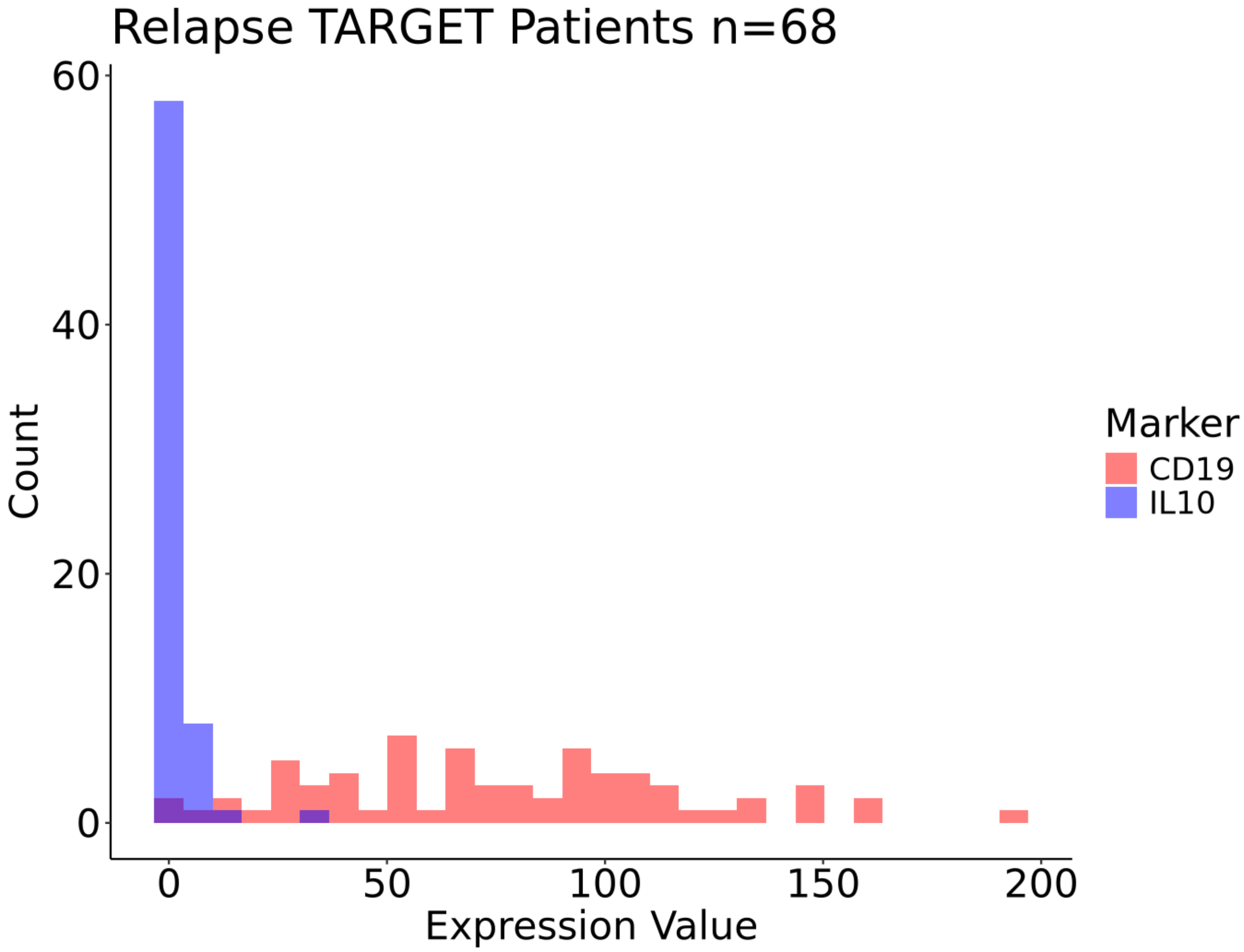

Histogram demonstrating RPKM expression of *CD19* and *IL10* from bulk RNA-sequencing data in the Therapeutically Applicable Research to Generate Effective Treatments (TARGET) Relapse Cohort (N=68).

Supplemental Figure 8.

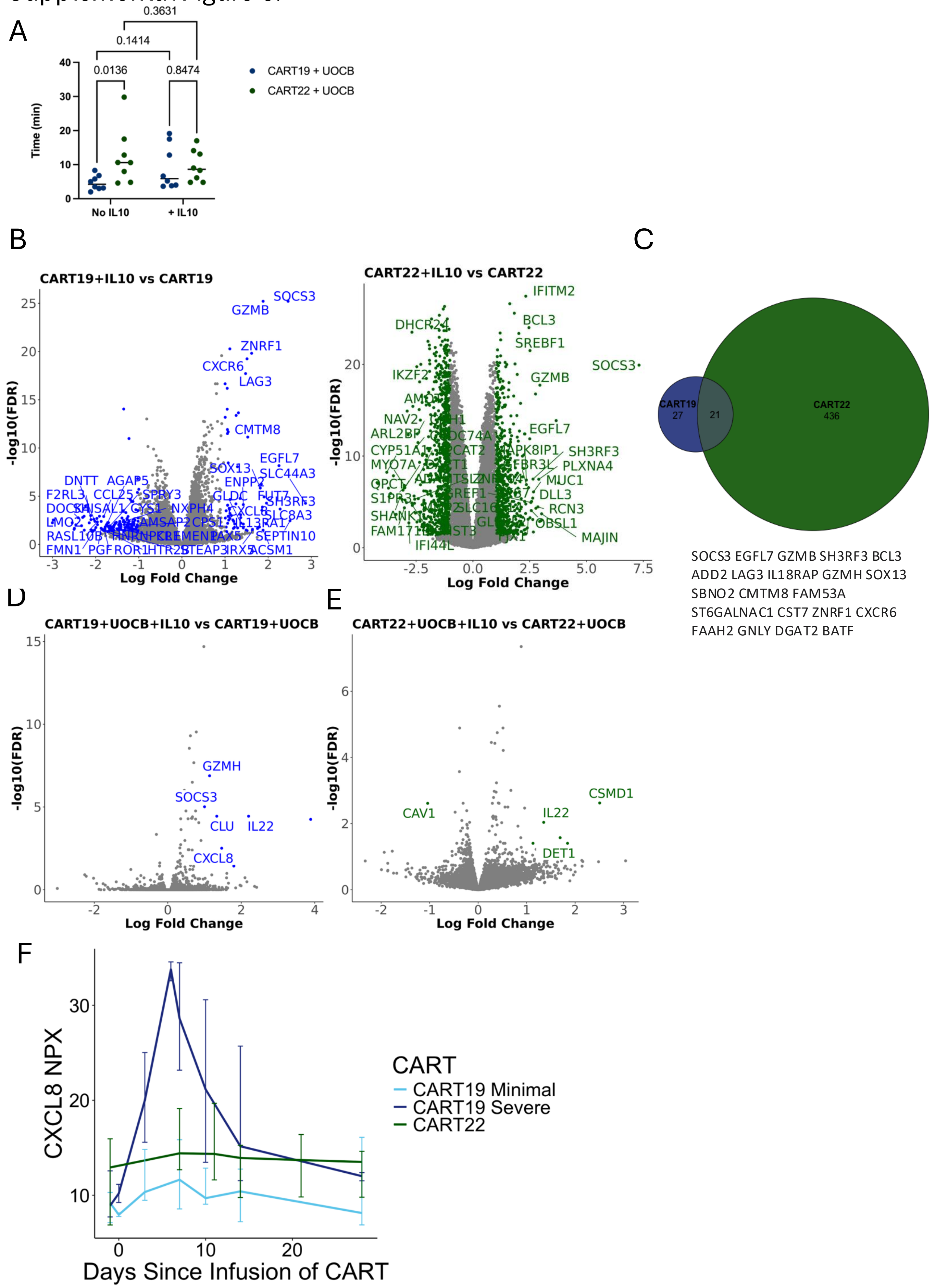

(A) Immune synapse measured between CART19 and CART22 and UOCB cells at an E:T ratio of 1:3 in the presence or absence of IL-10. Two donors measured in triplicate. **(B)** Differentially expressed genes (log fold change > 1, FDR < 0.05) between CART19 cells and CART22 cells with and without IL-10 added. **(C)** Euler diagram of overlap in genes between. 21 genes common between the two conditions are listed below. **(D)** DEG between CART19 cells co-cultured with UOCB cells at an E:T ratio of 1:8 in the presence and absence of IL10 (log fold change > 1, FDR < 0.05). **(E)** DEG between CART22 cells co-cultured with UOCB cells at an E:T ratio of 1:8 in the presence and absence of IL10 (log fold change > 1, FDR < 0.05). **(F)** Median CXCL8 (IL-8) NPX between CART19 minimal (light blue, N=13), CART19 severe (dark blue, N=10) and CART22 (green, N=17). Error bars represent IQR.

Supplemental Figure 9.

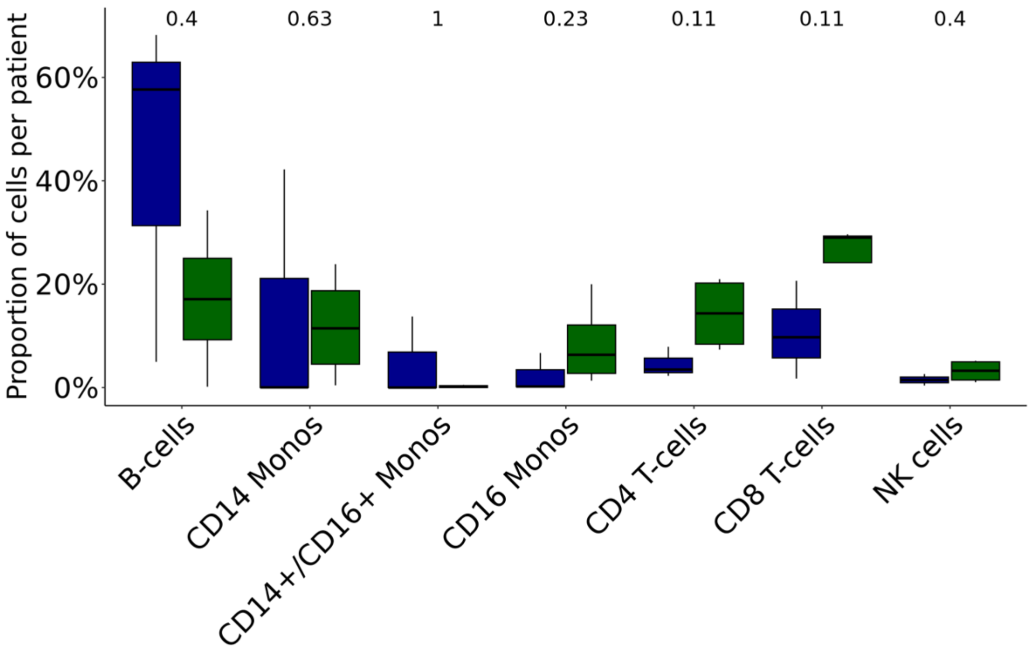

Proportion of cells in peripheral blood mononuclear cells between CART19 and CART22 patients. P-value calculated with Wilcoxon test.

Supplemental Figure 10.

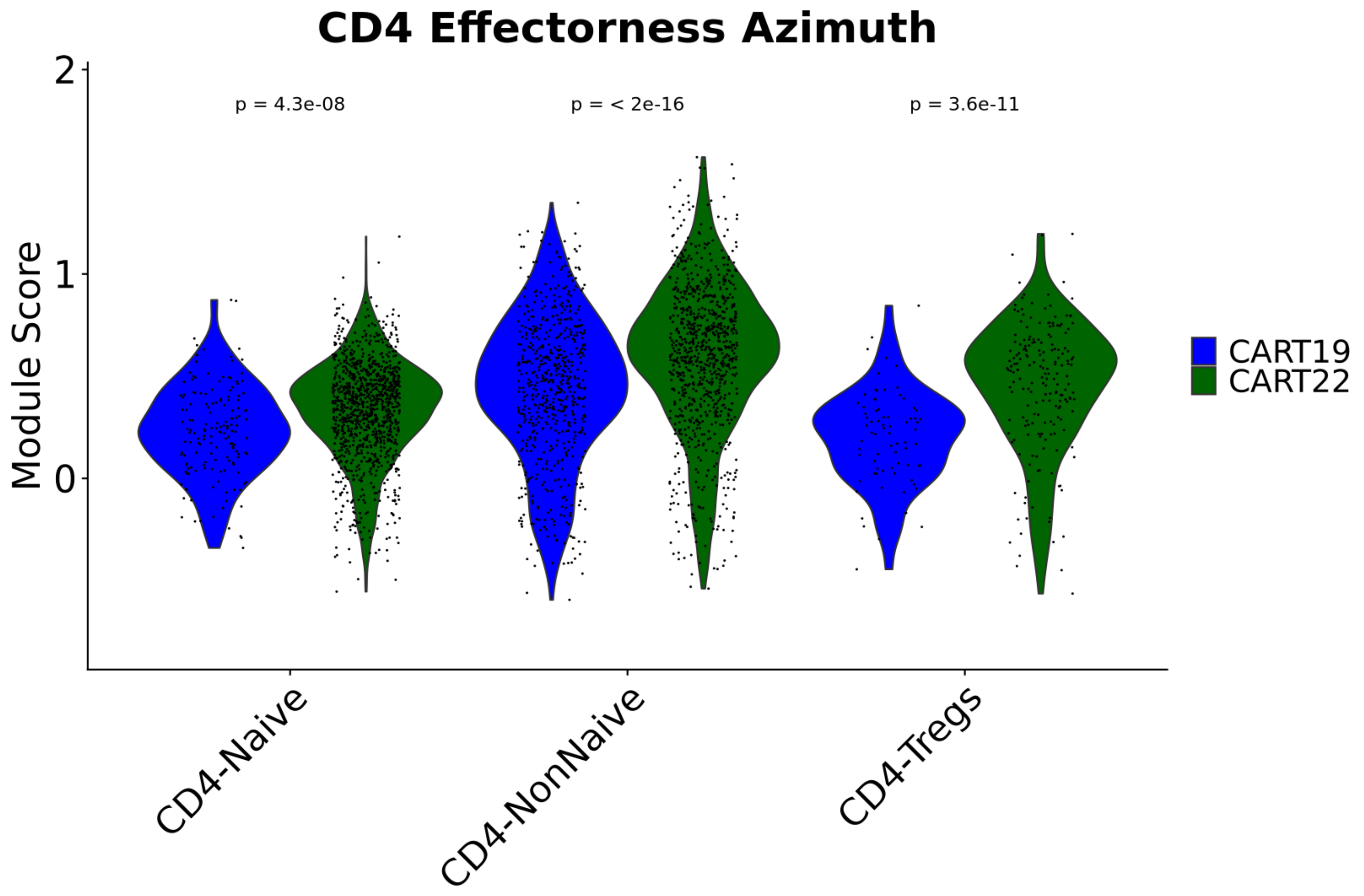

Violin plot of Azimuth CD4 effectorness score applied to CD4-naïve, CD4-non naïve and T-regulatory populations. P-values calculated with Wilcoxon test.

Supplemental Figure 11.

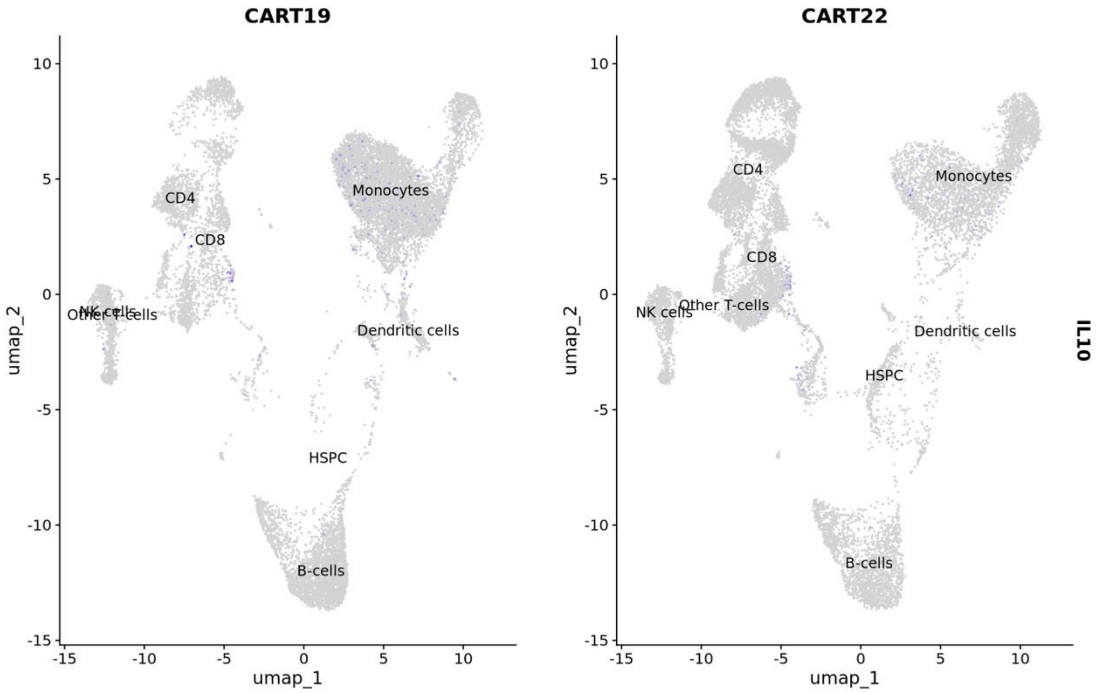

IL10 expression in peripheral blood mononuclear cells in CART19 and CART22 patients.

Supplemental Figure 12.

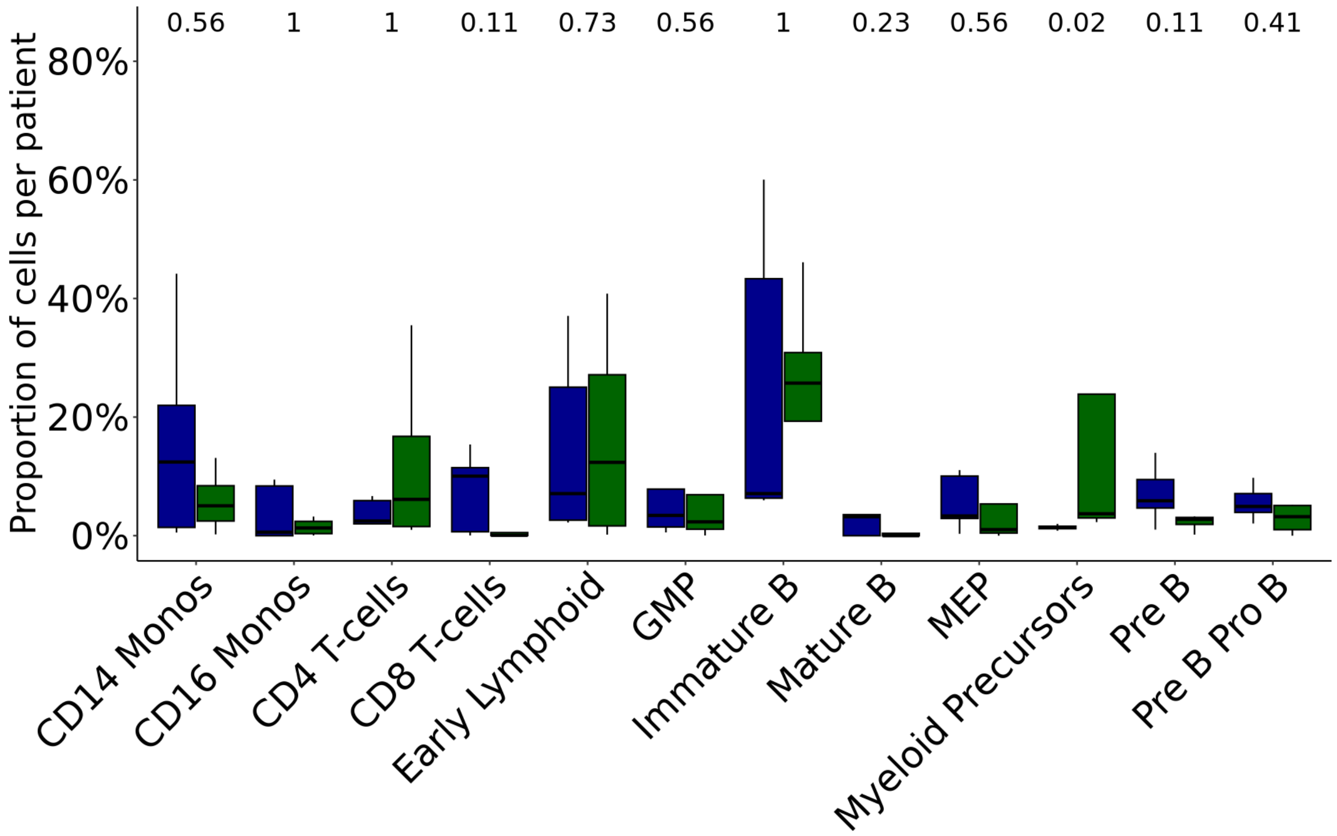

Box-plots of proportion of cell types in bone marrow between CART19 and CART22 patients. P-values calculated with Wilcoxon test.

Supplemental Figure 13.

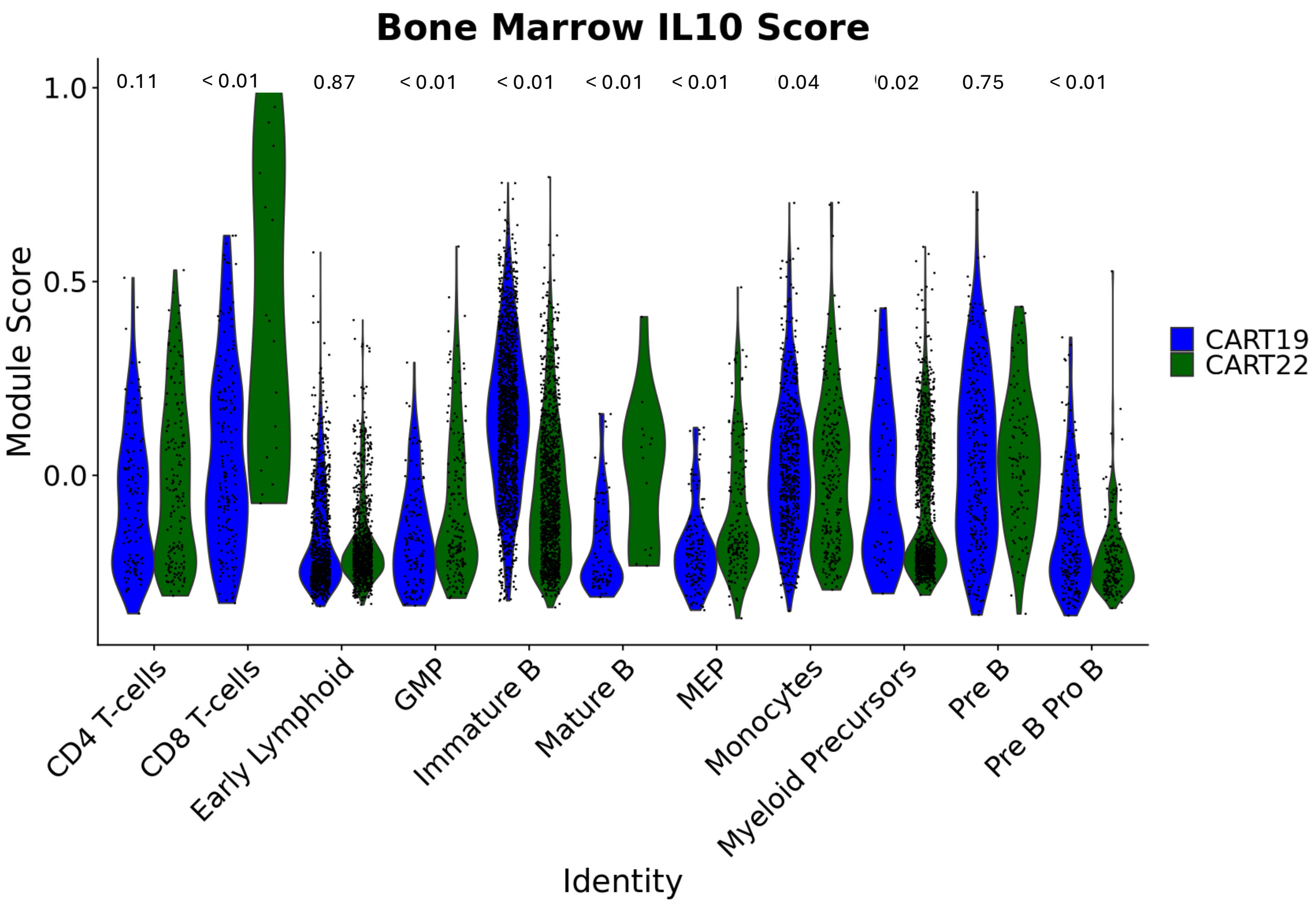

Violin plot of "IL-10 score" in scRNA-sequencing of bone marrow mononuclear cells (BMMC) populations between CART19 (blue) and CART22 (green) patients. P-value calculated with Wilcoxon test.

Supplemental Figure 14.

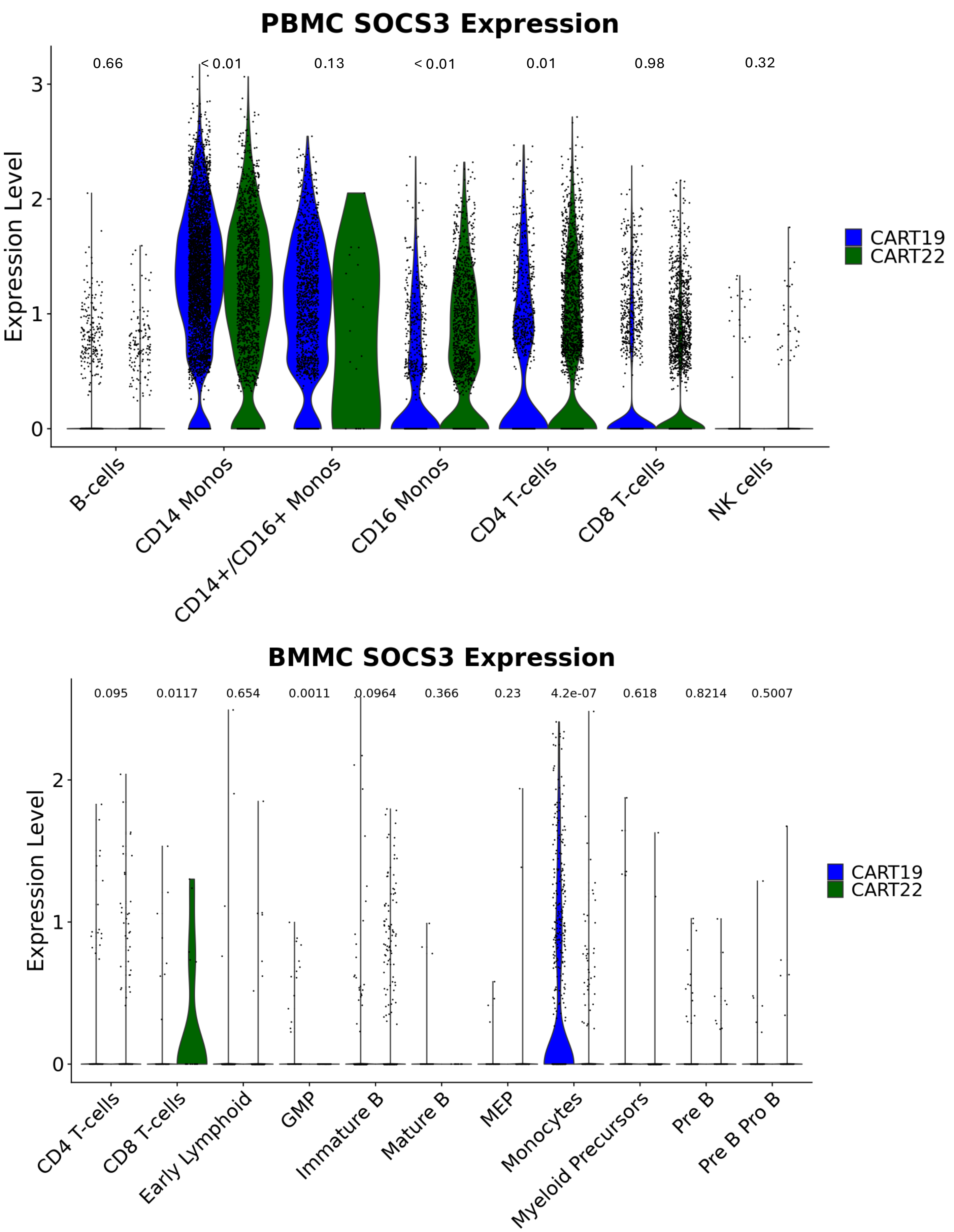

Violin plot of *SOCS3* expression in scRNA-sequencing PBMC and BMMC cellular populations in CART19 (blue) and CART22 (green) patients. P-value calculated with Wilcoxon test.

Supplemental Figure 15.

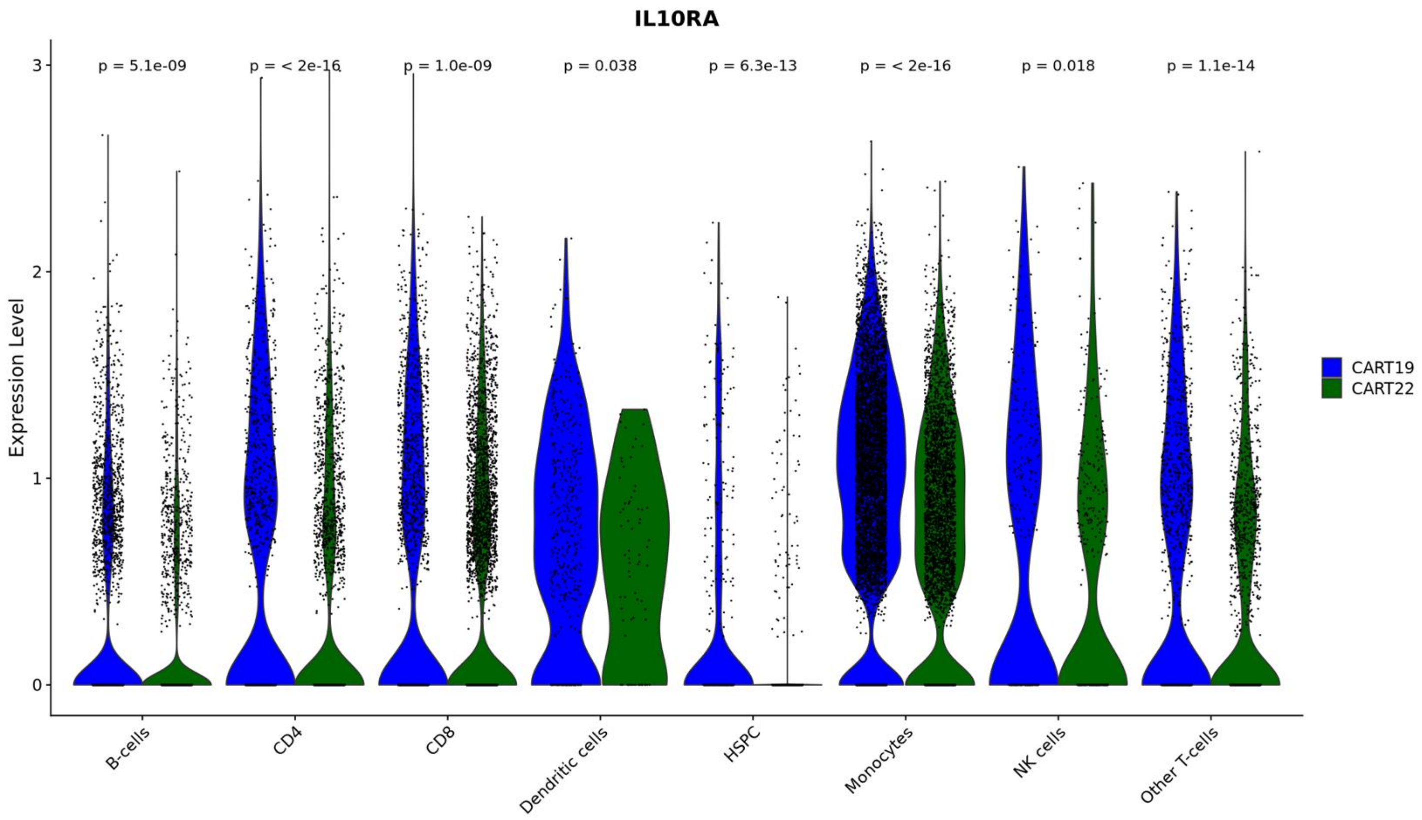

Violin plot of expression of *IL10RA* by PBMC cell type between CART19 and CART22 patients. P-value calculated with Wilcoxon test.
